# A general synthetic route to [Cu(X)(NHC)] (NHC = N-heterocyclic carbene, X = Cl, Br, I) complexes†
†Electronic supplementary information (ESI) available: Optimisation details and full characterisation data. CCDC 940850–940853. For ESI and crystallographic data in CIF or other electronic format see DOI: 10.1039/c3cc45488f



**DOI:** 10.1039/c3cc45488f

**Published:** 2013-10-01

**Authors:** Orlando Santoro, Alba Collado, Alexandra M. Z. Slawin, Steven P. Nolan, Catherine S. J. Cazin

**Affiliations:** a EaStCHEM School of Chemistry , University of St Andrews , St Andrews , KY16 9ST , UK . Email: cc111@st-andrews.ac.uk ; Fax: +44 (0)1334463808

## Abstract

A one-pot procedure for the synthesis of [Cu(X)(NHC)] (X = Cl, Br, I) is reported. The reaction is applicable to a wide range of saturated and unsaturated NHC ligands, is scalable and proceeds under mild conditions using technical grade solvents in air.

## Abstract 2

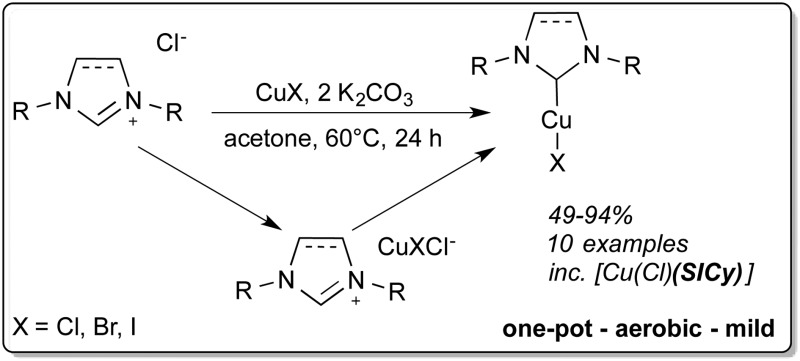
A series of complexes of the type [Cu(X)(NHC)] (X = I, Br, Cl, NHC = N-heterocyclic carbene) was synthesised using a one-pot, mild and user-friendly (aerobic, tech. grade solvents) procedure.

In transition metal-catalysed reactions the accessibility of the catalyst is one of the most important factors in dictating the usefulness of any catalytic method. The development of catalytic systems based on inexpensive metals such as iron^1^ and copper^2^ have gained increased attention during the last few years. In this context, N-heterocyclic carbene (NHC) copper species of the type [Cu(X)(NHC)] (X = Cl, Br, I) have shown to be efficient catalysts for several transformations,^3^ such as the reduction of carbonyl compounds,^4^ hydrosilylation^5^ and [3+2] cycloaddition of alkynes and azides.^6^ They can also be used as carbene transfer reagents to other transition metals such as gold or palladium.^7^ In addition, these systems have exhibited interesting biological activity as antitumour agents.^8^

The most common synthetic strategy to prepare [Cu(X)(NHC)] (X = Cl, Br, I) complexes is the reaction of a free carbene with a copper source.^9^ The free carbene can be isolated in the first instance or generated *in situ* (Scheme 1). The main drawbacks of this procedure are the need for an inert atmosphere and strictly anhydrous conditions as well as the use of strong and expensive bases.^5a,10^ In 2010 an improved procedure was developed by our group: the synthesis of several [Cu(Cl)(NHC)] complexes using Cu_2_O as a copper source in different solvents was reported (Scheme 1).^11^ This methodology allows the use of air-stable and economical starting materials and generates water as the only side-product. McQuade and co-workers have employed this system to validate their hypothesis that such syntheses could be performed in a continuous flow apparatus.^12^ In 2012, Jiang and co-workers reported the synthesis of some [Cu(Cl)(NHC)] by treatment of imidazolium salts with weak bases in the presence of a copper source.^13^ However, this protocol requires high temperature and environmentally unfriendly solvents such as 3-chloropyridine. Very recently, Cisnetti and co-workers showed that aqueous ammonia can promote the formation of Cu–NHC complexes from the corresponding imidazol(idin)ium salts.^14^

**Scheme 1:**
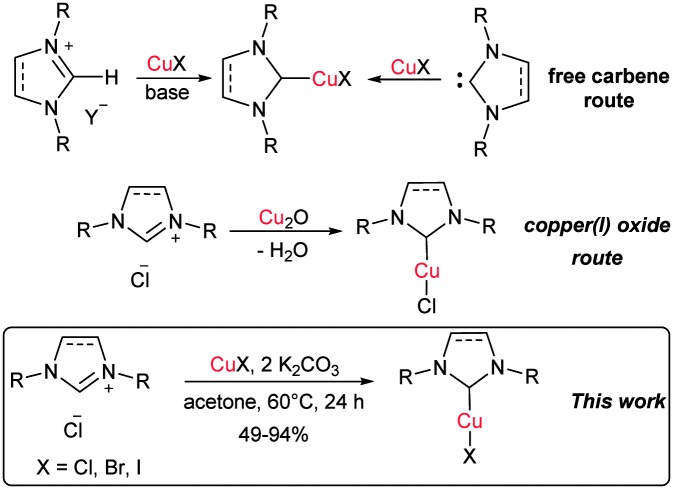
Synthetic routes to [Cu(X)(NHC)] complexes.

Despite these recent improvements, access to a general synthetic route leading to [Cu(X)(NHC)] complexes under milder and environmentally friendly conditions remains highly desirable.

Recently, one of us has reported a one-step methodology to prepare [Au(X)(NHC)] (X = Cl, Br, I) complexes, using imidazol(idin)ium salts, a gold source and a weak base. This protocol proceeds under mild conditions and has proven to be robust and general, as it is applicable to the synthesis of a wide range of NHC–Au complexes.^15,16^ Due to the great versatility of this protocol and the closely related chemistry of gold and copper, we were interested in extending this straightforward methodology to the preparation of [Cu(X)(NHC)] complexes (Fig. 1). Therefore we carried out the synthesis of [Cu(Cl)(IPr)] (**2a**) following the optimised conditions for gold, *e.g.*, treating, in air, IPr·HCl (**1a**) with CuCl in the presence of 1 equivalent of K_2_CO_3_, using technical grade acetone at 60 °C. After 24 h an encouraging 80% conversion to the desired complex **2a** was observed. By doubling the amount of base, **2a** was isolated in 92% yield (Scheme 2). Noteworthily, the reaction time can be reduced to 1 h by using 10 equivalents of base.

**Fig. 1:**
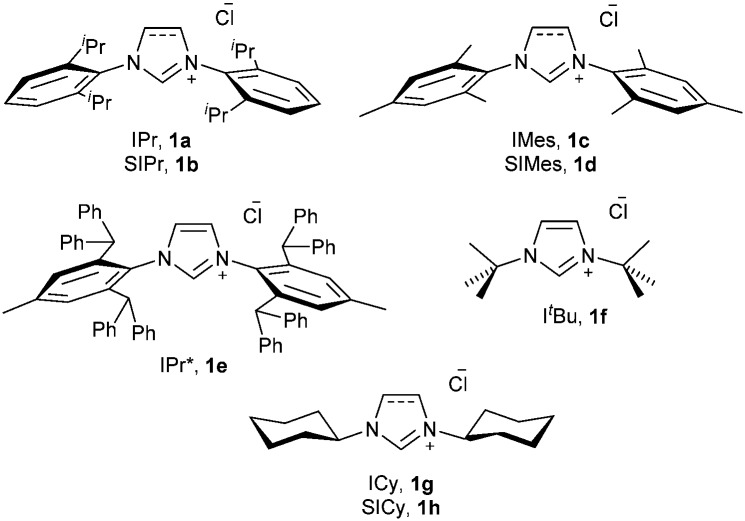
NHC·HCl salts used in this study.

**Scheme 2:**
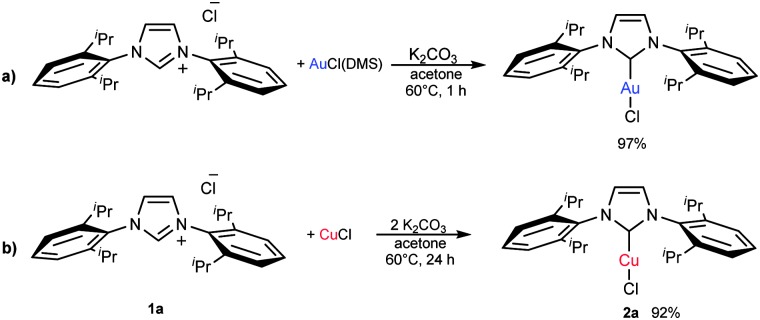
Optimised conditions for (a) [Au(Cl)(IPr)] and (b) **2a**.

After a brief optimisation of the reaction conditions (see ESI†), we found the best conditions for the synthesis of [Cu(Cl)(IPr)] are the ones used for the synthesis of [Au(Cl)(IPr)]. In order to evaluate the versatility of this protocol we carried out the reaction using different NHC ligands and copper salts under the optimised conditions. The results are summarised in Table 1.‡
‡General synthetic procedure: a vial was charged with NHC·HCl (100 mg), CuCl (1 equiv.), K_2_CO_3_ (2 equiv.). The resulting mixture was suspended in acetone (1.0 mL) and stirred at 60 °C for 24 h. After this time the mixture was filtered through silica. The pad of silica was washed with dichloromethane (3 × 1 mL). The solvent was concentrated and pentane (3 mL) was added affording the desired product that was washed with further portions of pentane (3 × 1 mL) and dried under vacuum. It should be noted that, although the reaction time can be significantly decreased by adding a large excess of base, all reactions were carried out with 2 equivalents in order to minimise waste.

**Table 1:** Scope of [Cu(Cl)(NHC)] synthesis^*a*^

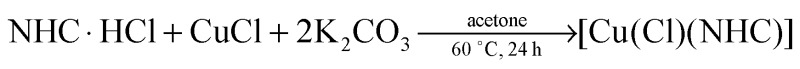			
Entry	NHC·HCl	Complex	Yield (%)
1	IPr, **1a**	[Cu(Cl)(IPr)], **2a**	92
2	SIPr, **1b**	[Cu(Cl)(SIPr)], **2b**	84
3	IMes, **1c**	[Cu(Cl)(IMes)], **2c**	76
4	SIMes, **1d**	[Cu(Cl)(SIMes)], **2d**	94
5	IPr*, **1e**	[Cu(Cl)(IPr*)], **2e**	70
6^*b*^	I^*t*^Bu, **1f**	[Cu(Cl)(I^*t*^Bu)], **2f**	55
7	ICy, **1g**	[Cu(Cl)(ICy)], **2g**	80
8^*b*^ ^,^ ^*c*^	SICy, **1h**	[Cu(Cl)(SICy)], **2h**	49

^*a*^Reaction conditions: NHC·HCl (100 mg), CuCl (1 equiv.), K_2_CO_3_ (2 equiv.), acetone, 60 °C, 24 h.

^*b*^Under Ar.

^*c*^In CH_2_Cl_2_.

This methodology is efficient for the synthesis of a wide variety of [Cu(Cl)(NHC)] complexes. All complexes were obtained with moderate to excellent yields and were characterised by ^1^H and ^13^C-{^1^H} NMR spectroscopy with data in agreement with the literature.^11,12,17^ The reaction was tested for the most common saturated and unsaturated NHC ligands bearing *N*-aryl moieties (Table 1, entries 1–4). This procedure allowed the preparation of NHC–copper(I) complexes in air using mild conditions, while previous methods required either an inert atmosphere (free carbene routes)^17*a*^ or harsher conditions (toluene or water reflux).^11^ Moreover, these reactions proceed cleanly and the formation of undesired side-products such as [Cu(NHC)_2_]^+^ was never observed, which is a common problem in the synthesis *via* the free carbene route.^17*a*^ The reaction involving the very bulky IPr* (**1e**) also succeeded (Table 1, entry 5), avoiding the microwave irradiation employed in its previously reported synthesis.^17*b*^ Copper complexes containing *N*-alkyl NHC ligands were also prepared (Table 1, entries 6–8). [Cu(Cl)(ICy)] (**2g**) was obtained in a higher yield (80%) than by previously reported routes (70–71%).^17*a*^ [Cu(Cl)(I^*t*^Bu)] (**2f**) was prepared under an Ar atmosphere as the complex is known to be air- and moisture-sensitive.^17*a*^ Although the reaction did not reach completion, the pure complex was isolated in 55% yield, avoiding the isolation of the free carbene or toluene reflux. In this case the formation of the [Cu(NHC)_2_]^+^ species was also not observed either.^11,17a^ A remarkable example is the synthesis of [Cu(Cl)(SICy)] (**2h**) (Table 1, entry 8). To the best of our knowledge, this complex has never been isolated in batch conditions and the only reported synthesis requires the use of a continuous flow system apparatus.^12^ Synthetic attempts following the Cu_2_O route led to the formation of a single species identified as the ketone 1,3-dicyclohexylimidazolidin-2-one.^11^ The optimal conditions to synthesise [Cu(Cl)(IPr)] led to an equimolar amount of **2h** and of the ketone when applied to SICy. For this reason, a new synthetic approach is needed for this complex. Gratifyingly, when using dichloromethane instead of acetone, the formation of the ketone was not observed and pure [Cu(Cl)(SICy)] was isolated as a white power. While this complex decomposes to a green gel when stored for extended periods under an Ar atmosphere, this methodology enabled its preparation.

As the nature of the halide has shown to influence the catalytic behaviour of [Cu(X)(NHC)],^17*a*^ the protocol described above was applied to access NHC–Cu derivatives containing different halides. Therefore, the reactions between IPr·HCl (**1a**) and CuBr and CuI in the presence of K_2_CO_3_ were carried out (Table 2). These reactions gave access to the corresponding bromide (**2i**) and iodide (**2j**) [Cu(X)(IPr)] complexes, respectively.

**Table 2:** Synthesis of [Cu(X)(IPr)] (X = Br, I) complexes^*a*^

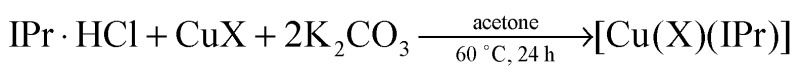				
Entry	NHC·HCl	CuX	Complex	Yield (%)
1	IPr·HCl, **1a**	CuBr	[Cu(Br)(IPr)], **2i**	88
2	IPr·HCl, **1a**	CuI	[Cu(I)(IPr)], **2j**	77

^*a*^Reaction conditions: NHC·HCl (100 mg), CuX (1 equiv.), K_2_CO_3_ (2 equiv.), acetone, 60 °C, 24 h.

In order to obtain more information about the mechanism of this transformation, the reaction of IPr·HCl and CuCl in the absence of base was performed. A new species was obtained in almost quantitative yield and characterised by ^1^H and ^13^C-{^1^H} NMR spectroscopy and by elemental analysis in addition to diffraction studies on a single crystal. The data revealed the product to be [IPrH][CuCl_2_] (**3a**),^18^ which consists of an imidazolium cation paired with a [CuCl_2_]^–^ counterion. **3a** is the copper counterpart of [IPrH][AuCl_2_], obtained in the reaction between IPr·HCl and [Au(Cl)(DMS)].^15^ As in the gold reaction, the formation of **3a** occurs at room temperature within the mixing time and can be further reacted with 2 equivalents of base in acetone, affording the final [Cu(Cl)(IPr)] complex (Scheme 3).

**Scheme 3:**
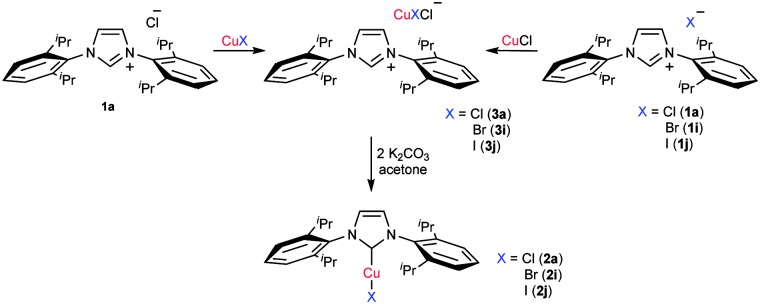
Formation and further reaction of the [IPrH][CuClX] species.

Next, the reactions between IPr·HCl and CuX (X = Br, I) were studied and two new [IPrH][CuClX] species (**3i** and **3j**) were obtained. The same complexes can also be obtained by the reaction between the corresponding IPr·HX salts and CuCl. Subsequent treatment of **3i** and **3j** with K_2_CO_3_ afforded the corresponding [Cu(X)(IPr)] **2i** and **2j** (Scheme 3). It should be noted that the formation of the analogous chloride complex **2a** was never observed. A new intermediate species, [IPrH][CuBrI] (**3k**), was prepared by treatment of CuI with IPr·HBr. Further treatment of **3k** with K_2_CO_3_ afforded [Cu(I)(IPr)] as a single product. This trend, also observed for gold,^15^ can be explained by considering the halide *trans* effect series: I ≫ Br > Cl.^19^ On the basis of this evidence we postulated that the halide exerting a higher *trans* effect labilises the bond *trans* to it and then this halide remains coordinated to the copper in the final complex. In addition, the lattice energies for the formation of the KX salts (KCl > KBr > KI) are in agreement with the observed reactivity.

Due to the interesting nature of these species, [SIPrH][CuCl_2_] (**3b**), [IMesH][CuCl_2_] (**3c**), [SIMesH][CuCl_2_] (**3d**) and [ICyH][CuCl_2_] (**3g**) analogues were synthesised and their structures have been confirmed by X-ray diffraction studies on single crystals.^20^ Also in this case, the treatment of these species with K_2_CO_3_ under the reaction conditions led to the formation of the corresponding complexes. These results reinforce the hypothesis that the [(NHC)H][CuCl_2_] species are the actual intermediates in this transformation.

Finally, we were interested in testing the efficiency of this protocol in the synthesis of [Cu(X)(IPr)] complexes on a larger scale (Table 3). It should be noted that the reaction time increased to several days when 2 equiv. of base were used and, in order to provide a practical synthetic protocol, an additional equivalent of K_2_CO_3_ was used.

**Table 3:** Larger-scale reactions^*a*^

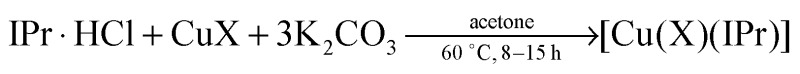					
Entry	IPr·HCl (g)	CuX (g)	Complex	Time (h)	Yield (%)
1	1.00	CuCl (0.22)	[Cu(Cl)(IPr)], **2a**	15	90
2	1.00	CuBr (0.33)	[Cu(Br)(IPr)], **2i**	8	91
3	0.88	CuI (0.36)	[Cu(I)(IPr)], **2j**	8	85

^*a*^Reaction conditions: acetone, 60 °C, 1 equiv. of IPr·HCl, 1 equiv. of CuX, 3 equiv. of K_2_CO_3_.

In summary a one-pot general procedure for the synthesis of [Cu(X)(NHC)] (X = Cl, Br, I) under mild and aerobic conditions was developed. This synthetic method was shown to proceed *via* a [(NHC)H][CuX_2_] intermediate species. A wide variety of NHC ligands, as well as halide sources, were efficiently used. Moreover, this protocol allowed for the synthesis of [Cu(Cl)(SICy)] which has not been previously achieved under batch conditions. Further synthetic and theoretical studies dealing with the mechanism of the reaction are currently ongoing.

The authors gratefully acknowledge the Royal Society (University Research Fellowship to C.S.J.C.), EaStCHEM School of Chemistry, the ERC (Advanced Investigator Award-FUNCAT and PoC project GOLDCAT) and the EPSRC for funding. S.P.N. is a Royal Society Wolfson Research Merit Award holder. We also thank Mr Adrián Gómez-Suárez for early synthetic contributions and discussions.
