# Supplementary material for: A general synthetic route to [Cu(X)(NHC)] (NHC = N-heterocyclic carbene, X = Cl, Br, I) complexes
Source: Chem Commun (Camb). 2013 Oct 1;49(89):10435. doi: 10.1039/c3cc45488f (PMC4155829; doi:10.1039/c3cc45488f)
Supplement: Supplementary file 1 [file CC-049-C3CC45488F-s001.pdf]

Supporting Information

for

A general synthetic route to [Cu(X)(NHC)] (NHC = *N*-heterocyclic carbene, X =Cl, Br, I) complexes

*Orlando Santoro, Alba Collado, Alexandra M. Z. Slawin, Steven P. Nolan and Catherine S. J. Cazin**

EaStCHEM School of Chemistry, University of St Andrews, St Andrews, KY16 9ST, UK.

cc111@st-andrews.ac.uk

Table of Contents

General considerations S3

Synthesis of the [Cu(X)(NHC)] complexes S3

Screening of the reaction conditions S3

General Procedure S3

Small scale: S3

Large scale S4

Synthesis of [Cu(Cl)(IPr)] (**2a**) S4

Synthesis of [Cu(Cl)(SIPr)] (**2b**) S5

Synthesis of [Cu(Cl)(IMes)] (**2c**) S5

Synthesis of [Cu(Cl)(SIMes)] (**2d**) S6

Synthesis of [Cu(Cl)(IPr*)] (**2e**) S6

Synthesis of [Cu(Cl)(I^{t}Bu)] (**2f**) S7

Synthesis of [Cu(Cl)(ICy)] (**2g**) S7

Synthesis of [Cu(Cl)(SICy)] (**2h**) S8

Synthesis of [Cu(Br)(IPr)] (**2i**) S9

Synthesis of [Cu(I)(IPr)] (**2j**) S9

Synthesis of the [(NHC)H][CuXY] salts (**3a-3k**) S10

General Procedure S10

Synthesis of [IPrH][$CuCl_2$] (**3a**) S10

Synthesis of [SIPrH][$CuCl_2$] (**3b**) S11

Synthesis of [IMesH][$CuCl_2$] (**3c**) S12

Synthesis of [SIMesH][$CuCl_2$] (**3d**) S12

Synthesis of [ICyH][$CuCl_2$] (**3g**) S13

Synthesis of [IPrH][CuClBr] (**3i**) S14

Synthesis of [IPrH][CuClI] (**3j**) S14

Synthesis of [IPrH][CuBrI] (**3k**) S15

^{1}H and ^{13}C-{1H} NMR spectra S16

Crystal data and Structure refinement S34

General considerations

All reactions were carried under air and technical grade solvent were used unless otherwise stated. K_2CO_3 and $KHCO_3$ were used as received without further purification. ^1H, and ^{13}C-{^1H} Nuclear Magnetic Resonance (NMR) spectra were recorded on a Bruker ADVANCE 300 MHz and Bruker ADVANCE 400 MHz spectrometer using the residual solvent peak as reference ($CHCl_3$: δ_H = 7.26 ppm, δ_C = 77.16 ppm, CH_2Cl_2: δ_H = 5.32 ppm, δ_C = 53.84 ppm) at 298K. Elemental analyses were performed at London Metropolitan University 166-220, Holloway Road, London, N7 8DB.

Synthesis of the [Cu(X)(NHC)] complexes

Screening of the reaction conditions

Entry	Base (equiv.)	Solvent	T (°C)	Time (h)	Conversion (%)[a]
1	K_2CO_3 (1.5)	acetone	rt	24	75
2	K_2CO_3 (2.0)	acetone	rt	24	84
3	K_2CO_3 (1.0)	acetone	60	24	80
**4**	**K_2CO_3 (2.0)**	**acetone**	**60**	**24**	**>99**
**5**	**K_2CO_3 (10)**	**acetone**	**60**	**1**	**>99**
6	$KHCO_3$ (2.0)	acetone	60	24	95
7	K_2CO_3 (2.0)	THF	60	24	80

[a]Conversion determined by ^1H NMR analysis.

General Procedure

Small scale:

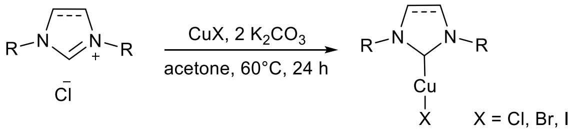

A vial was charged with NHC·HCl (1.0 equiv.), CuX (1.0 equiv.) and K_2CO_3 (2.0 equiv.). The mixture was dissolved in acetone (1.0 mL) and stirred at 60 °C for 24 hours. The solution was then filtered through silica which was washed with dichloromethane (3 x 1.0 mL). The solvent was concentrated under vacuum and pentane (3.0 mL) was added thereby precipitating the desired product that was washed with further portions of pentane (3 x 1.0 mL) and dried under vacuum.

Large scale: a round bottom flask equipped with a condenser was charged with IPr·HCl (1.0 equiv.), CuX (X = Cl, Br, I, 1.0 equiv.) and K_2CO_3 (3.0 equiv.). The mixture was dissolved in acetone and stirred for 8-15 h at 60 °C. The same work-up as the small scale procedure was carried out.

Synthesis of [Cu(Cl)(IPr)] (2a)

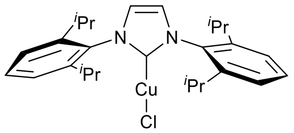

Reaction between IPr·HCl (**1a**) (100 mg, 0.23 mmol), CuCl (23 mg, 0.23 mmol) and K_2CO_3 (66 mg, 0.46 mmol) led to the isolation of **2a** as a white solid in 92% isolated yield (103 mg, 0.21 mmol).

^{1}H NMR (400 MHz, $CDCl_3$, 298 K): δ = 1.23 (d, $^3J_{H-H}$ = 6.9 Hz, 12H, CH-C*H₃*), 1.30 (d, $^3J_{H-H}$ = 6.9 Hz, 12H, CH-CH_3), 2.56 (sept, $^3J_{H-H}$ = 6.9 Hz, 4H, C*H*-CH_3), 7.13 (s, 2H, H^4 and H^5), 7.29 (d, $^3J_{H-H}$ = 7.8 Hz, 4H, C*H* phenyl), 7.49 (t, $^3J_{H-H}$ = 7.8 Hz, 2H, C*H* phenyl).

^{13}C-{^{1}H} NMR (75 MHz, $CDCl_3$, 298 K): δ = 24.0 (s, CH-*C*H_3), 24.9 (s, CH-*C*H_3), 28.8 (s, *C*H-CH_3), 123.3 (s, C^{IV} Ar), 124.3 (s, *C*H Ar), 130.7 (s, C^4 and C^5), 134.5 (s, C^{IV} Ar), 145.7 (s, *C*H Ar), 180.6 (s, C^2).

Elem. Anal. Calcd. for $C_{27}H_{37}ClCuN_2$: C, 66.37; H, 7.63, N, 5.73. Found: C, 66.62, H, 7.31, N, 5.84

Synthesis of [Cu(Cl)(SIPr)] (2b)

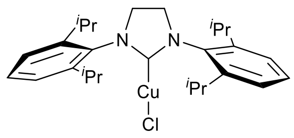

Reaction between SIPr·HCl (**1b**) (100 mg, 0.23 mmol), CuCl (23 mg, 0.23 mmol) and K_2CO_3 (66 mg, 0.46 mmol) led to the isolation of **2b** as a white solid in 84% isolated yield (94 mg, 0.19 mmol).

^{1}H NMR (400 MHz, $CDCl_3$, 298 K): δ = 1.34 (d, $^3J_{H-H}$ = 7.0 Hz, 12H, CH-CH_3), 1.36 (d, $^3J_{H-H}$ = 7.0 Hz, 12H, CH-CH_3), 3.06 (sept, $^3J_{H-H}$ = 6.9 Hz, 4H, C*H*-CH_3), 4.01 (s, 4H, H^4 and H^5), 7.24 (d, $^3J_{H-H}$ = 7.8 Hz, 4H, C*H* phenyl), 7.39 (t, $^3J_{H-H}$ = 7.8 Hz, 2H, C*H* phenyl).

^{13}C-{^{1}H} NMR (75 MHz, $CDCl_3$, 298 K): δ = 24.0 (s, CH-*C*H_3), 25.6 (s, CH-*C*H_3), 29.0 (s, *C*H-CH_3), 53.8 (s, C^4 and C^5), 124.6 (s, *C*H Ar), 130.0 (s, C^{IV} *C*H Ar), 134.5 (s, C^{IV} Ar), 146.7 (s, *C*H Ar), 203.1 (s, C^2).

Synthesis of [Cu(Cl)(IMes)] (2c)

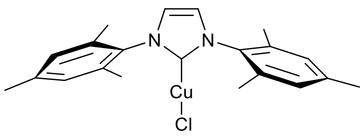

Reaction between IMes·HCl (**1c**) (100 mg, 0.29 mmol), CuCl (29 mg, 0.29 mmol) and K_2CO_3 (80 mg, 0.58 mmol) led to the isolation of **2c** as a white solid in 76% isolated yield (89 mg, 0.22 mmol).

^{1}H NMR (400 MHz, $CDCl_3$, 298 K): δ = 2.10 (s, 12H, CH_3), 2.34 (s, 6H, CH_3), 6.99 (s, 4H, C*H* phenyl), 7.05 (s, 2H, H^4 and H^5).

^{13}C-{^{1}H} NMR (75 MHz, $CDCl_3$, 298 K): δ = 17.9 (s, *C*H_3), 21.2 (s, *C*H_3), 122.4 (s, C^{IV} Ar), 129.6 (s, *C*H Ar), 134.7 (s, C^4 and C^5), 135.2 (s, C^{IV} Ar), 139.6 (s, C^{IV} Ar), 179.1 (s, C^2).

Synthesis of [Cu(Cl)(SIMes)] (2d)

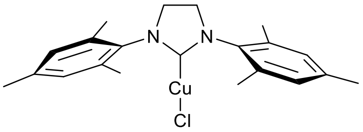

Reaction between SIMes·HCl (**1d**) (100 mg, 0.29 mmol), CuCl (29 mg, 0.29 mmol) and K_2CO_3 (80 mg, 0.58 mmol) led to the isolation of **2d** as a white solid in 94% isolated yield (110 mg, 0.27 mmol).

^{1}H NMR (400 MHz, $CDCl_3$, 298 K): δ = 2.29 (s, 6H, CH_3), 2.30 (s, 12H, CH_3), 3.93 (s, 2H, H^4 and H^5), 6.93 (s, 4H, C*H* phenyl).

^{13}C-{^{1}H} NMR (75 MHz, $CDCl_3$, 298 K): δ = 18.1 (s, *C*H$_3$), 21.1 (s, *C*H$_3$), 51.0 (s, C^4 and C^5), 129.8 (s, *C*H Ar), 135.1 (s, C^{IV} Ar), 135.5 (s, C^{IV} Ar), 138.6 (s, C^{IV} Ar), 202.5 (s, C^2).

Synthesis of [Cu(Cl)(IPr*)] (2e)

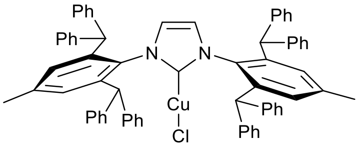

Reaction between IPr*·HCl (**1e**) (100 mg, 0.10 mmol), CuCl (9.90 mg, 0.10 mmol) and K_2CO_3 (27.6 mg, 0.20 mmol) led to the isolation of **2e** as a white solid in 70% isolated yield (71 mg, 0.07 mmol).

^{1}H NMR (400 MHz, $CDCl_3$, 298 K): δ = 2.23 (s, 6H, CH_3), 5.21 (s, 4H, C*H*Ph$_2$), 5.83 (s, 2H, H^4 and H^5), 6.86 (s, 4H, C*H* phenyl), 6.90-6.92 (m, 8H, C*H* phenyl), 7.03 (m, 8H, C*H* aryl), 7.15-7.21 (m, 24H, C*H* aryl).

^{13}C-{^{1}H} NMR (75 MHz, $CDCl_3$, 298 K): δ = 21.9 (s, *C*H$_3$), 51.3 (s, *C*HPh$_2$), 123.3 (s, C^4 and C^5), 126.7 (s, *C*H Ar), 126.8 (s, *C*H Ar), 128.5 (s, *C*H Ar), 128.7 (s, *C*H Ar), 129.5 (s, *C*H Ar), 129.7 (s, *C*H Ar), 130.3 (s, *C*H Ar), 134.3 (s, C^{IV} Ar), 140.1 (s, C^{IV} Ar), 141.0 (s, C^{IV} Ar), 142.4 (s, C^{IV} Ar), 143.2 (s, C^{IV} Ar), 180.4 (s, C^2).

Synthesis of [Cu(Cl)(I^{t}Bu)] (2f)

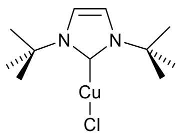

Reaction (carried out under Ar atmosphere) between I^{t}Bu·HCl (**1f**) (100 mg, 0.37 mmol), CuCl (36.6 mg, 0.37 mmol) and K_2CO_3 (99.4 mg, 0.74 mmol) led to the isolation of **2f** as a white solid in 55% isolated yield (47 mg, 0.21 mmol).

^{1}H NMR (400 MHz, $CDCl_3$, 298 K): δ = 1.75 (s, 18H, CH_3), 7.03 (s, 2H, H^4 and H^5).

^{13}C-{^{1}H} NMR (75 MHz, $CDCl_3$, 298 K): δ = 32.1 (s, CH$_3$), 116.7 (s, C^4 and C^5), 172.8 (s, C^2).

Synthesis of [Cu(Cl)(ICy)] (2g)

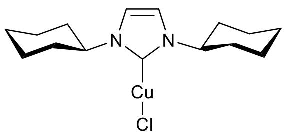

Reaction between ICy·HCl (**1g**) (100 mg, 0.37 mmol), CuCl (36.7 mg, 0.37 mmol) and K_2CO_3 (99.4 mg, 0.74 mmol) led to the isolation of **2g** as a white solid in 80% isolated yield (98 mg, 0.30 mmol).

^{1}H NMR (400 MHz, $CDCl_3$, 298 K): δ = 1.20 (m, 2H, CH_2), 1.40 (m, 4H, CH_2), 1.56-1.71 (m, 6H, CH_2), 1.84 (m, 4H, CH_2), 2.00 (m, 4H, CH_2), 4.24 (m, 2H, CH), 6.91 (s, 2H, H^4 and H^5).

^{13}C-{^{1}H} NMR (75 MHz, $CDCl_3$, 298 K): δ = 25.0 (s, CH$_2$), 25.4 (s, CH$_2$), 34.7 (s, CH$_2$), 61.1 (s, CH), 117.5 (s, C^4 and C^5), 173.3 (s, C^2).

Synthesis of [Cu(Cl)(SICy)] (2h)

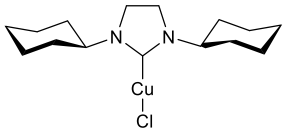

Under Ar, a vial was charged with SICy·HCl (**1h**) (100 mg, 0.37 mmol, 1.0 equiv.), CuCl (36.6 mg, 0.37 mmol, 1.0 equiv.) and K_2CO_3 (99.4 mg, 0.74 mmol, 2.0 equiv.) The mixture was dissolved in dry dichloromethane (1.0 mL) and stirred at 60°C for 24 hours. The solution was then filtered through silica which was washed with dichloromethane (3 x 1 mL). Evaporation under reduced pressure of the volatiles led to the isolation of **2h** as a white solid in 49% isolated yield (60 mg, 0.19 mmol).

^{1}H NMR (400 MHz, $CDCl_3$, 298 K): δ = 1.04 (m, 2H, C*H*$_2$), 1.29 (m, 4H, C*H*$_2$), 1.43 (m, 4H, C*H*$_2$), 1.61 (m, 2H, C*H*$_2$), 1.76 (m, 8H, C*H*$_2$), 3.45 (s, $^3J_{H\text{-}H}$ = 12.0 Hz, 4H, H^4 and H^5), 3.77 (m, 2H, C*H*).

^{13}C-{^{1}H} NMR (75 MHz, $CDCl_3$, 298 K): δ = 25.2 (s, *C*H$_2$), 25.3 (s, *C*H$_2$), 31.9 (s, *C*H$_2$), 44.2 (s, C^4 and C^5), 59.6 (s, *C*H), 197.3 (s, C^2).

Synthesis of [Cu(Br)(IPr)] (2i)

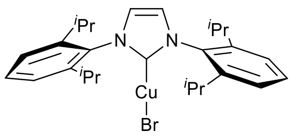

Reaction between IPr·HCl (**1a**) (100 mg, 0.23 mmol), CuBr (33 mg, 0.23 mmol) and K_2CO_3 (66 mg, 0.46 mmol) led to the isolation of **2i** as a white solid in 88% isolated yield (106 mg, 0.20 mmol).

^{1}H NMR (400 MHz, $CDCl_3$, 298 K): δ = 1.22 (d, $^3J_{H-H}$ = 6.9 Hz, 12H, CH-C*H*$_3$), 1.30 (d, $^3J_{H-H}$ = 6.9 Hz, 12H, CH-C*H*$_3$), 2.56 (sept, $^3J_{H-H}$ = 6.9 Hz, 4H, C*H*-CH_3), 7.14 (s, 2H, H^4 and H^5), 7.29 (d, $^3J_{H-H}$ = 7.8 Hz, 4H, C*H* phenyl), 7.48 (t, $^3J_{H-H}$ = 7.8 Hz, 2H, C*H* phenyl).

^{13}C-{^{1}H} NMR (75 MHz, $CDCl_3$, 298 K): δ = 24.0 (s, CH-*C*H_3), 24.9 (s, CH-*C*H_3), 28.8 (s,*C*H-CH_3), 123.2 (s, C^{IV} Ar), 124.3 (s, *C*H Ar), 130.6 (s, C^4 and C^5), 134.4 (s, C^{IV} Ar), 145.6 (s, *C*H Ar), 181.2 (s, C^2).

Synthesis of [Cu(I)(IPr)] (2j)

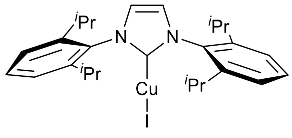

Reaction between IPr·HCl (**1a**) (100 mg, 0.23 mmol), CuI (44 mg, 0.23 mmol) and K_2CO_3 (66 mg, 0.46 mmol) led to the isolation of **2j** as a white solid in 77% isolated yield (104 mg, 0.18 mmol).

^{1}H NMR (400 MHz, $CDCl_3$, 298 K): δ = 1.23 (d, $^3J_{H-H}$ = 6.9 Hz, 12H, CH-C*H*$_3$), 1.30 (d, $^3J_{H-H}$ = 6.9 Hz, 12H, CH-C*H*$_3$), 2.57 (sept, $^3J_{H-H}$ = 6.9 Hz, 4H, C*H*-CH_3), 7.14 (s, 2H, H^4 and H^5), 7.30 (d, $^3J_{H-H}$ = 7.8 Hz, 4H, C*H* phenyl), 7.49 (t, $^3J_{H-H}$ = 7.8 Hz, 2H, C*H* phenyl).

^{13}C-{^{1}H} NMR (75 MHz, $CDCl_3$, 298 K): δ = 24.0 (s, CH-CH_3), 25.0 (s, CH-CH_3), 28.8 (s, *C*H-CH_3), 123.2 (s, C^{IV} Ar), 124.3 (s, *C*H Ar), 130.7 (s, C^4 and C^5), 134.3 (s, C^{IV} Ar), 145.7 (s, *C*H Ar), 183.0 (s, C^2).

Synthesis of the [(NHC)H][CuXY] salts (3a-3k)

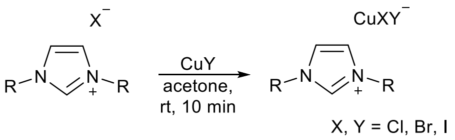

General Procedure

NHC·HX and CuY (X, Y = Cl, Br, I) were charged in a vial equipped with a magnetic stirring bar. The final solids mixture was dissolved in acetone (3.0 mL) and stirred for 10 minutes at room temperature. After this time the solvent was removed under reduced pressure affording the product.

Synthesis of [IPrH][$CuCl_2$] (3a)

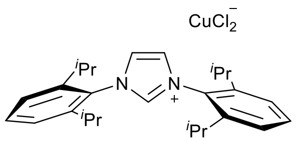

Reaction between IPr·HCl (**1a**) (300 mg, 0.70 mmol) and CuCl (69 mg, 0.70 mmol) led to the isolation of **3a** as a white solid in 95% isolated yield (350 mg, 0.66 mmol).

^{1}H NMR (400 MHz, CD_2Cl_2, 298 K): δ = 1.22 (d, $^{3}J_{H-H}$ = 7.1 Hz, 12H, CH-C*H₃*), 1.28 (d, $^{3}J_{H-H}$ = 7.1 Hz, 12H, CH-C*H₃*), 2.39 (sept, $^{3}J_{H-H}$ = 7.1 Hz, 4H, C*H*-CH_3), 7.39 (d, $^{3}J_{H-H}$ = 7.8 Hz, 4H, C*H* phenyl), 7.62 (t, $^{3}J_{H-H}$ = 7.8 Hz, 2H, C*H* phenyl), 7.79 (s, 2H, H^4 and H^5) 10.00 (s, 1H, H^2).

^{13}C-{^{1}H} NMR (75 MHz, CD_2Cl_2, 298 K): δ = 23.8 (s, CH-CH_3), 24.7 (s, CH-CH_3), 29.5 (s, *C*H-CH_3), 125.1 (s, *C*H Ar), 126.2 (s, *C*H Ar), 130.1 (s, C^{IV} Ar), 136.5 (s, C^4 and C^5), 139.2 (s, C^2), 145.3 (s, C^{IV} Ar).

Elem. Anal.: Calcd. for $C_{27}H_{37}Cl_2CuN_2$: C, 61.88; H, 7.12, N, 5.35. Found: C, 61.96, H, 7.15, N, 5.41

Synthesis of [SIPrH][CuCl$_2$] (3b)

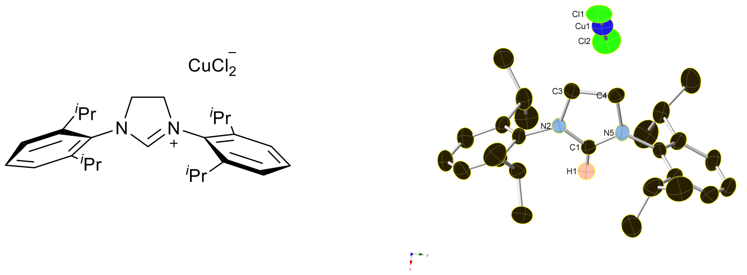

Reaction between SIPr·HCl (**1b**) (100 mg, 0.23 mmol), CuCl (23 mg, 0.23 mmol) led to the isolation of **3b** as a white solid in 87% isolated yield (109 mg, 0.20 mmol). Crystals suitable for X-ray diffraction were grown by slow diffusion of pentane in a saturated solution of **3b** in dichloromethane.

^{1}H NMR (400 MHz, CD_2Cl_2, 298 K): δ = 1.26 (d, $^3J_{H\text{-}H}$ = 6.9 Hz, 12H, CH-CH_3), 1.39 (d, $^3J_{H\text{-}H}$ = 6.9 Hz, 12H, CH-CH_3), 3.00 (sept, $^3J_{H\text{-}H}$ = 7.0 Hz, 4H, CH-CH$_3$), 4.59 (s, 4H, H^4 and H^5), 7.32 (d, $^3J_{H\text{-}H}$ = 7.9 Hz, 4H, CH phenyl), 7.52 (t, $^3J_{H\text{-}H}$ = 7.9 Hz, 2H, CH phenyl), 8.63 (s, 1H, H^2).

^{13}C-{^{1}H} NMR (75 MHz, CD_2Cl_2, 298 K): δ = 24.1 (s, CH-CH$_3$), 25.5 (s, CH-CH$_3$), 29.6 (s, CH-CH$_3$), 54.6 (s, C^4 and C^5), 125.5 (s, CH Ar), 129.4 (s, C^{IV} CH Ar), 132.1 (s, C^{IV} Ar), 146.5 (s, CH Ar), 159.2 (s, C^2).

Elem. Anal.: Calcd. for $C_{27}H_{39}Cl_2CuN_2$: C, 61.65; H, 7.47, N, 5.33. Found: C, 61.75, H, 7.57, N, 5.40

Synthesis of [IMesH][CuCl$_2$] (3c)

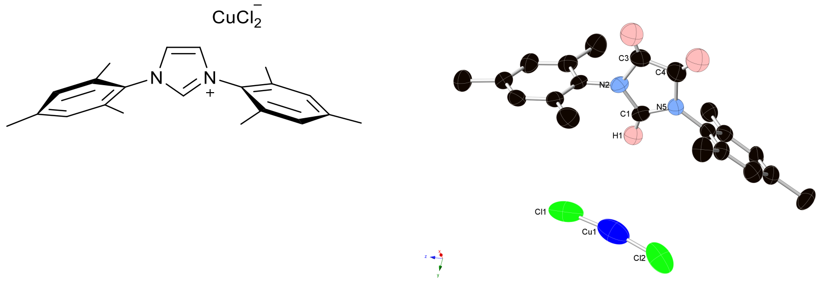

Reaction between IMes·HCl (**1c**) (100 mg, 0.29 mmol), CuCl (29 mg, 0.29 mmol) led to the isolation of **3c** as a white solid in 95% isolated yield (112 mg, 0.28 mmol). Crystals suitable for X-ray diffraction were grown by slow diffusion of pentane in a saturated solution of **3c** in dichloromethane.

^{1}H NMR (400 MHz, CD_2Cl_2, 298 K): δ = 2.16 (s, 12H, C*H$_3$*), 2.39 (s, 6H, C*H$_3$*), 7.11 (s, 4H, C*H* phenyl), 7.65 (s, 2H, H^4 and H^5), 9.55 (s, 1H, H^2).

^{13}C-{^{1}H} NMR (75 MHz, CD2Cl2, 298 K): δ = 17.8 (s, *C*H$_3$), 21.3 (s, *C*H$_3$), 125.2 (s, C^4 and C^5), 130.3 (s, *C*H Ar), 130.7 (s, C^{IV} Ar), 134.5 (s, C^2), 138.0 (s, C^{IV} Ar), 142.2 (s, C^{IV} Ar).

Elem. Anal.: Calcd. for $C_{21}H_{25}Cl_2CuN_2$: C, 57.34; H, 5.73, N, 6.37. Found: C, 57.44, H, 5.82, N, 6.46

Synthesis of [SIMesH][CuCl$_2$] (3d)

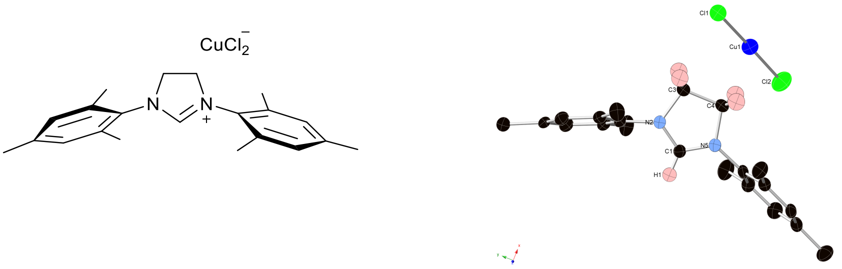

Reaction between SIMes·HCl (100 mg, 0.29 mmol), CuCl (29 mg, 0.29 mmol) led to the isolation of **3d** as a white solid in 94% isolated yield (110 mg, 0.27 mmol). Crystals suitable for X-ray diffraction were grown by slow diffusion of pentane in a saturated solution of **3d** in dichloromethane.

^{1}H NMR (400 MHz, CD_2Cl_2, 298 K): δ = 2.33 (s, 6H, C*H$_3$*), 2.39 (s, 12H, C*H$_3$*), 4.53 (s, 4H, H^4 and H^5), 7.04 (s, 4H, C*H* phenyl), 8.47 (s, 1H, H^2).

$^{13}C-\{^1H\}$ NMR (75 MHz, CD_2Cl_2, 298 K): δ = 18.3 (s, CH_3), 21.2 (s, CH_3), 52.0 (s, C^4 and C^5), 130.2 (s, C^{IV} Ar), 130.5 (s, *C*H Ar), 135.3 (s, C^{IV} Ar), 141.5 (s, C^{IV} Ar), 159.4 (s, C^2).

Elem. Anal.: Calcd. for $C_{21}H_{27}Cl_2CuN_2$: C, 57.08; H, 6.16, N, 6.34. Found: C, 57.16, H, 6.08, N, 6.40

Synthesis of $[ICyH][CuCl_2]$ (3g)

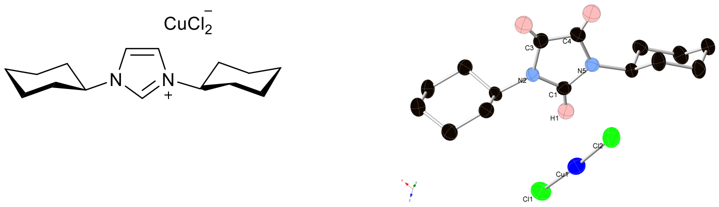

Reaction between ICy·HCl (**1g**) (100 mg, 0.37 mmol), CuCl (36.7 mg, 0.37 mmol) led to the isolation of **3g** as a white solid in 95% isolated yield (130 mg, 0.35 mmol). Crystals suitable for X-ray diffraction were grown by slow diffusion of pentane in a saturated solution of **3g** in dichloromethane.

^1H NMR (400 MHz, $CDCl_3$, 298 K): δ = 1.29 (m, 2H, CH_2), 1.53 (m, 4H, CH_2), 1.69-1.76 (m, 8H, CH_2), 1.94 (m, 4H, CH_2), 2.27 (m, 4H, CH_2), 4.52 (m, 2H, C*H*), 7.30 (s, 2H, H^4 and H^5), 10.29 (s, 1H, H^2).

$^{13}C-\{^1H\}$ NMR (75 MHz, $CDCl_3$, 298 K): δ = 24.6 (s, CH_2), 24.9 (s, CH_2), 33.6 (s, CH_2), 60.2 (s, *C*H), 120.3 (s, C^4 and C^5), 133.6 (s, C^2).

Elem. Anal.: Calcd. for $C_{15}H_{25}Cl_2CuN_2$: C, 48.98; H, 6.85, N, 7.62. Found: C, 48.97, H, 6.73, N, 7.67.

Synthesis of [IPrH][CuClBr] (3i)

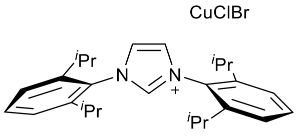

Reaction between IPr·HCl (300 mg, 0.70 mmol) and CuBr (100 mg, 0.70 mmol) led to the isolation of **3i** as a white solid in 93% isolated yield (339 mg, 0.65 mmol).

^{1}H NMR (400 MHz, CD_2Cl_2, 298 K): δ = 1.24 (d, $^3J_{H\text{-}H}$ = 7.1 Hz, 12H, CH-C*H₃*), 1.30 (d, $^3J_{H\text{-}H}$ = 7.1 Hz, 12H, CH-CH_3), 2.40 (sept, $^3J_{H\text{-}H}$ = 7.1 Hz, 4H, C*H*-CH_3), 7.41 (d, $^3J_{H\text{-}H}$ = 7.8 Hz, 4H, C*H* phenyl), 7.65 (t, $^3J_{H\text{-}H}$ = 7.8 Hz, 2H, C*H* phenyl), 7.80 (s, 2H, H^4 and H^5), 9.16 (s, 1H, H^2).

^{13}C-{^{1}H} NMR (75 MHz, CD_2Cl_2, 298 K): δ = 24.0 (s, CH-CH_3), 24.8 (s, CH-CH_3), 29.5 (s, *C*H-CH_3), 125.3 (s, *C*H Ar), 126.5 (s, *C*H Ar), 129.7 (s, C^{IV} Ar), 132.9 (s, C^4 and C^5), 138.0 (s, C^2),145.3 (s, C^{IV} Ar).

Elem. Anal.: Calcd. for $C_{27}H_{37}BrClCuN_2$: C, 57.04; H, 6.56, N, 4.93. Found: C, 56.91, H, 6.63, N, 5.01

Synthesis of [IPrH][CuClI] (3j)

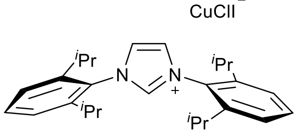

Reaction between IPr·HCl (300 mg, 0.70 mmol) and CuI (130 mg, 0.70 mmol) led to the isolation of **3j** as a white solid in 95% isolated yield (400 mg, 0.66 mmol).

^{1}H NMR (400 MHz, CD_2Cl_2, 298 K): δ = 1.23 (d, $^3J_{H\text{-}H}$ = 7.1 Hz, 12H, CH-CH_3), 1.30 (d, $^3J_{H\text{-}H}$ = 7.1 Hz, 12H, CH-CH_3), 2.41 (sept, $^3J_{H\text{-}H}$ = 7.1 Hz, 4H, C*H*-CH_3 isopropyl), 7.42 (d, $^3J_{H\text{-}H}$ = 7.8 Hz, 4H, C*H* phenyl), 7.63 (t, $^3J_{H\text{-}H}$ = 7.8 Hz, 2H, C*H* phenyl), 7.84 (d, 2H, H^4 and H^5), 9.10 (t, 1H, H^2).

^{13}C-{^{1}H} NMR (75 MHz, CD_2Cl_2, 298 K): δ = 24.0 (s, CH-CH_3), 24.8 (s, CH-CH_3), 29.5 (s, CH-CH_3), 125.4 (s, CH Ar), 126.6 (s, CH Ar), 129.7 (s, C^{IV} Ar), 132.9 (s, C^4 and C^5), 137.6 (s, C^2), 145.3 (s, C^{IV} Ar).

Elem. Anal.: Calcd. for $C_{27}H_{37}ClCuIN_2$: C, 52.69; H, 6.06, N, 4.55. Found: C, 52.78, H, 6.07, N, 4.61

Synthesis of [IPrH][CuBrI] (3k)

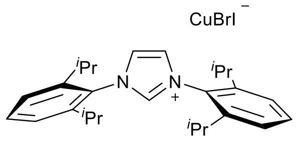

Reaction between IPr·HBr (300 mg, 0.70 mmol) and CuI (120 mg, 0.70 mmol) led to the isolation of **3k** as a white solid in 94% isolated yield (410 mg, 0.66 mmol).

^{1}H NMR (400 MHz, CD_2Cl_2, 298 K): δ = 1.24 (d, $^{3}J_{H\text{-}H}$ = 7.1 Hz, 12H, CH-CH_3), 1.32 (d, $^{3}J_{H\text{-}H}$ = 7.1 Hz, 12H, CH-CH_3), 2.41 (sept, $^{3}J_{H\text{-}H}$ = 7.1 Hz, 4H, CH-CH_3), 7.44 (d, $^{3}J_{H\text{-}H}$ = 7.8 Hz, 4H, CH phenyl), 7.67 (t, $^{3}J_{H\text{-}H}$ = 7.8 Hz, 2H, CH phenyl), 7.83 (s, 2H, H^4 and H^5), 8.87 (s, 1H, H^2).

^{13}C-{^{1}H} NMR (75 MHz, CD_2Cl_2, 298 K): δ = 24.0 (s, CH-CH_3), 24.8 (s, CH-CH_3), 29.5 (s, CH-CH_3), 125.3 (s, CH Ar), 126.7 (s, CH Ar), 129.7 (s, C^{IV} Ar), 132.9 (s, C^4 and C^5), 137.4 (s, C^2,145.3 (s, C^{IV} Ar).

Elem. Anal.: Calcd. for $C_{27}H_{37}BrCuIN_2$: C, 49.14; H, 5.65, N, 4.24. Found: C, 49.04, H, 5.59, N, 4.27

^{1}H and ^{13}C-{1H} NMR spectra

[Cu(Cl)(IPr)] **2a**, ^{1}H NMR, CDCl$_3$, 298K and ^{13}C-{^{1}H} NMR, CDCl$_3$, 298 K

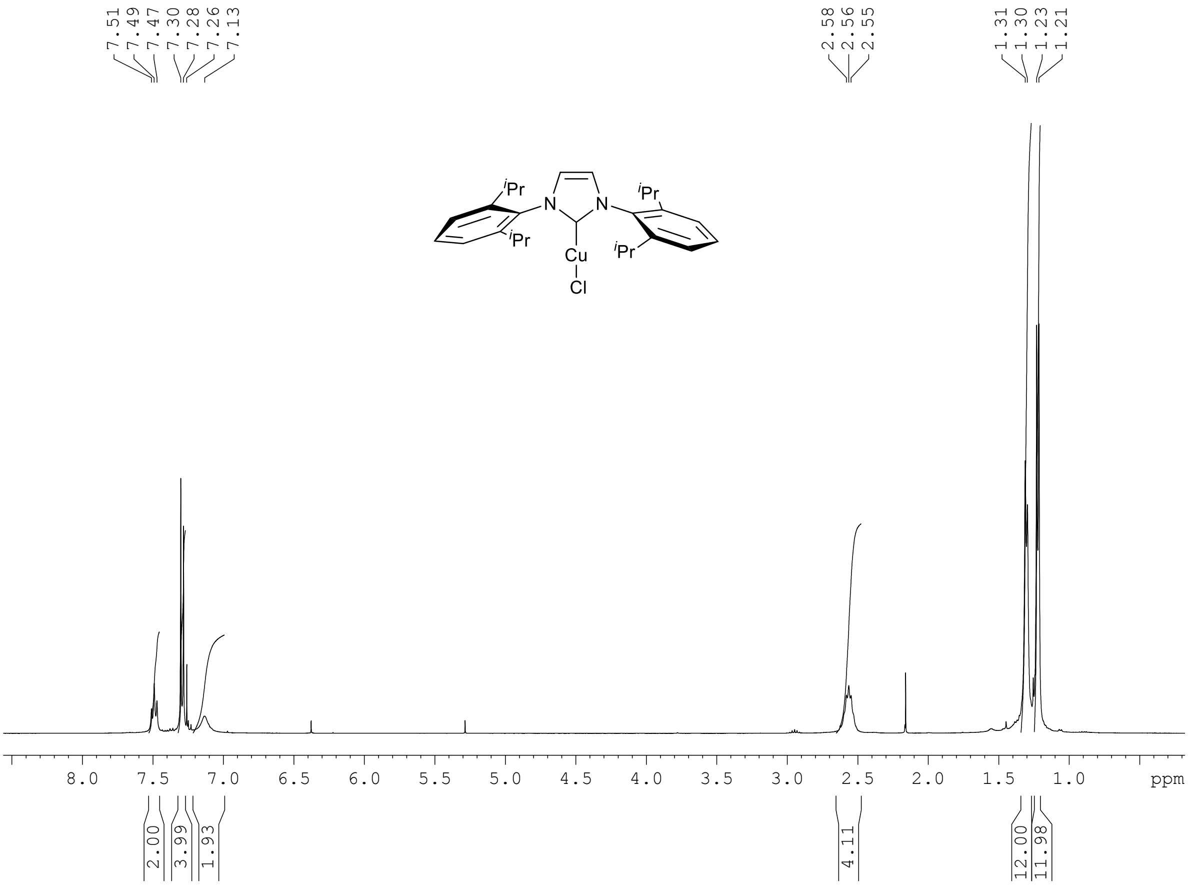

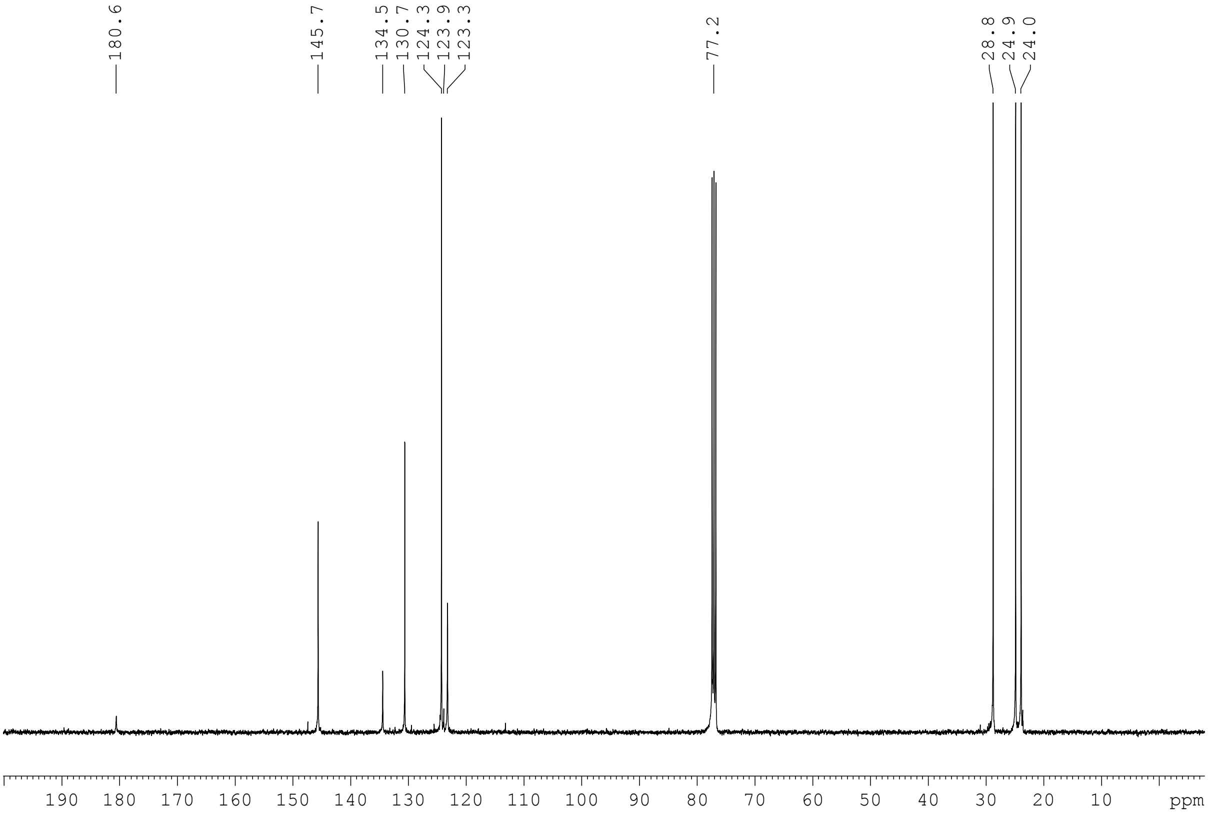

[Cu(Cl)(SIPr)] **2b**, ^{1}H NMR, $CDCl_3$, 298K and ^{13}C-{^{1}H} NMR, $CDCl_3$, 298 K

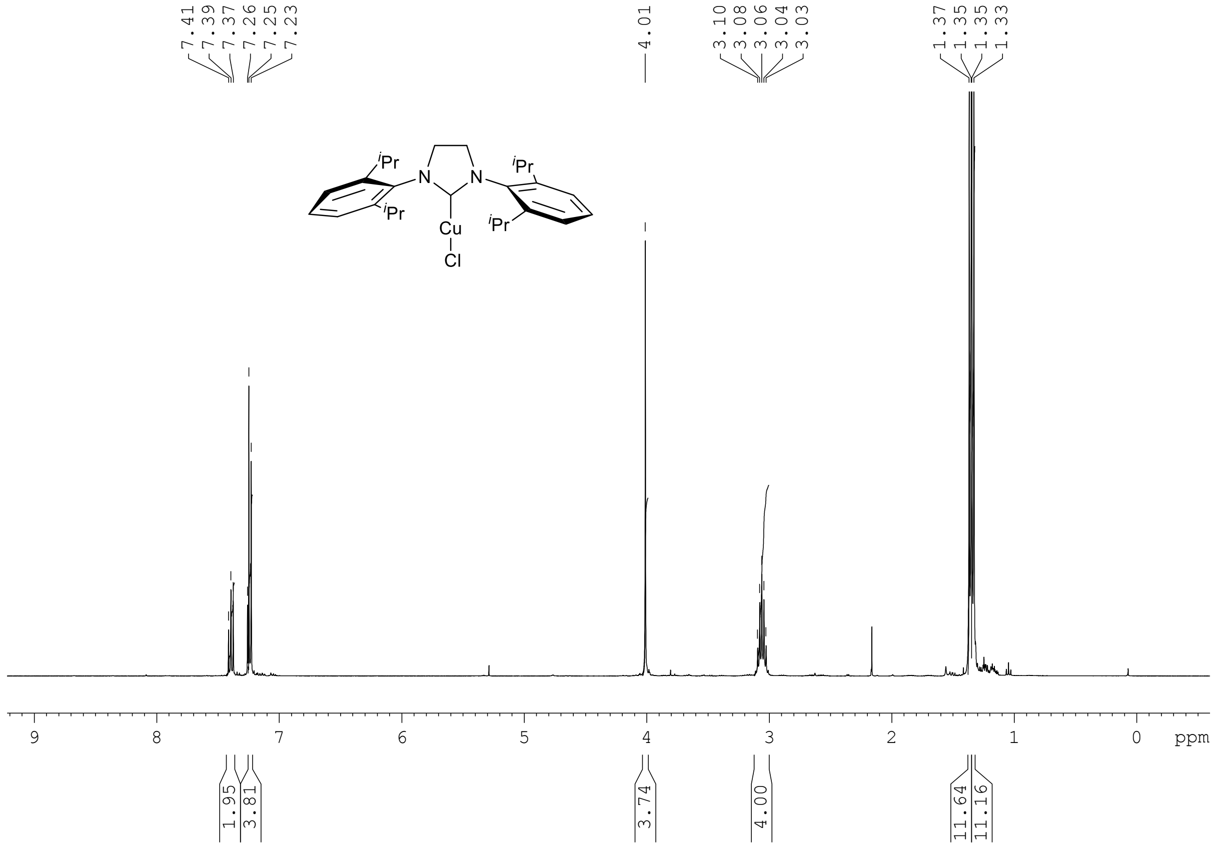

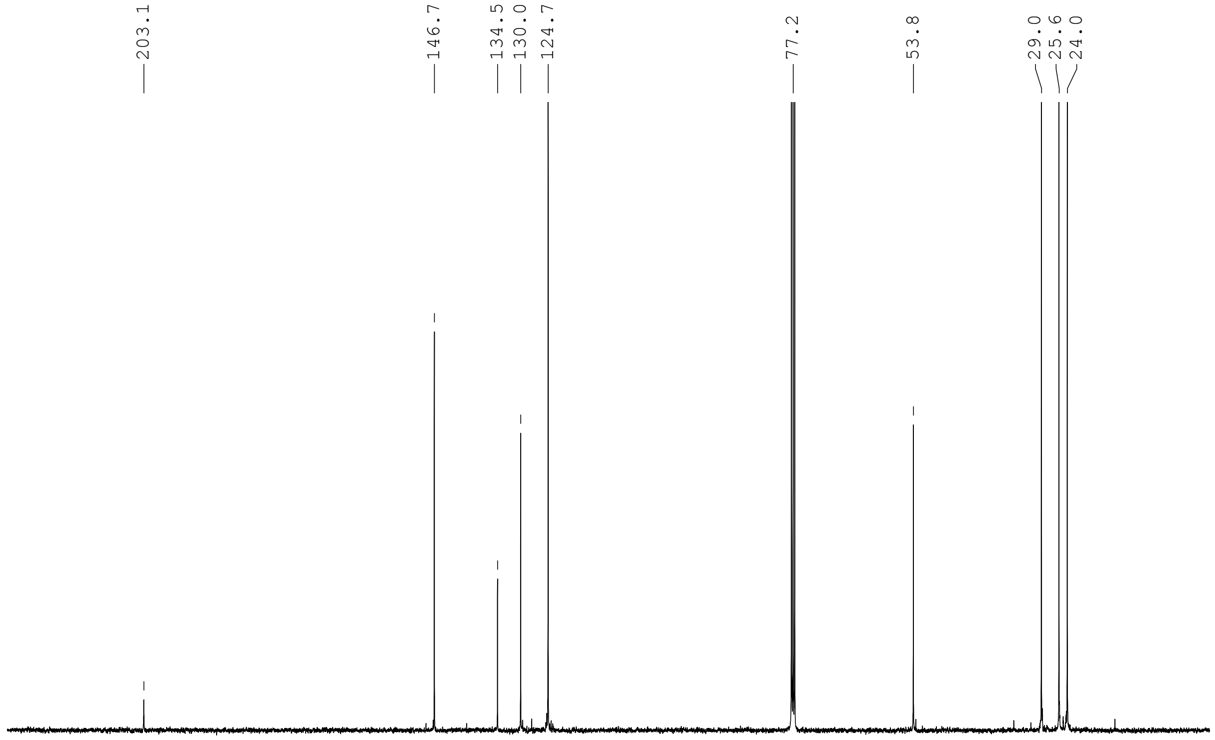

[Cu(Cl)(IMes)] **2c**, ^{1}H NMR, $CDCl_3$, 298K and ^{13}C-{^{1}H} NMR, $CDCl_3$, 298 K

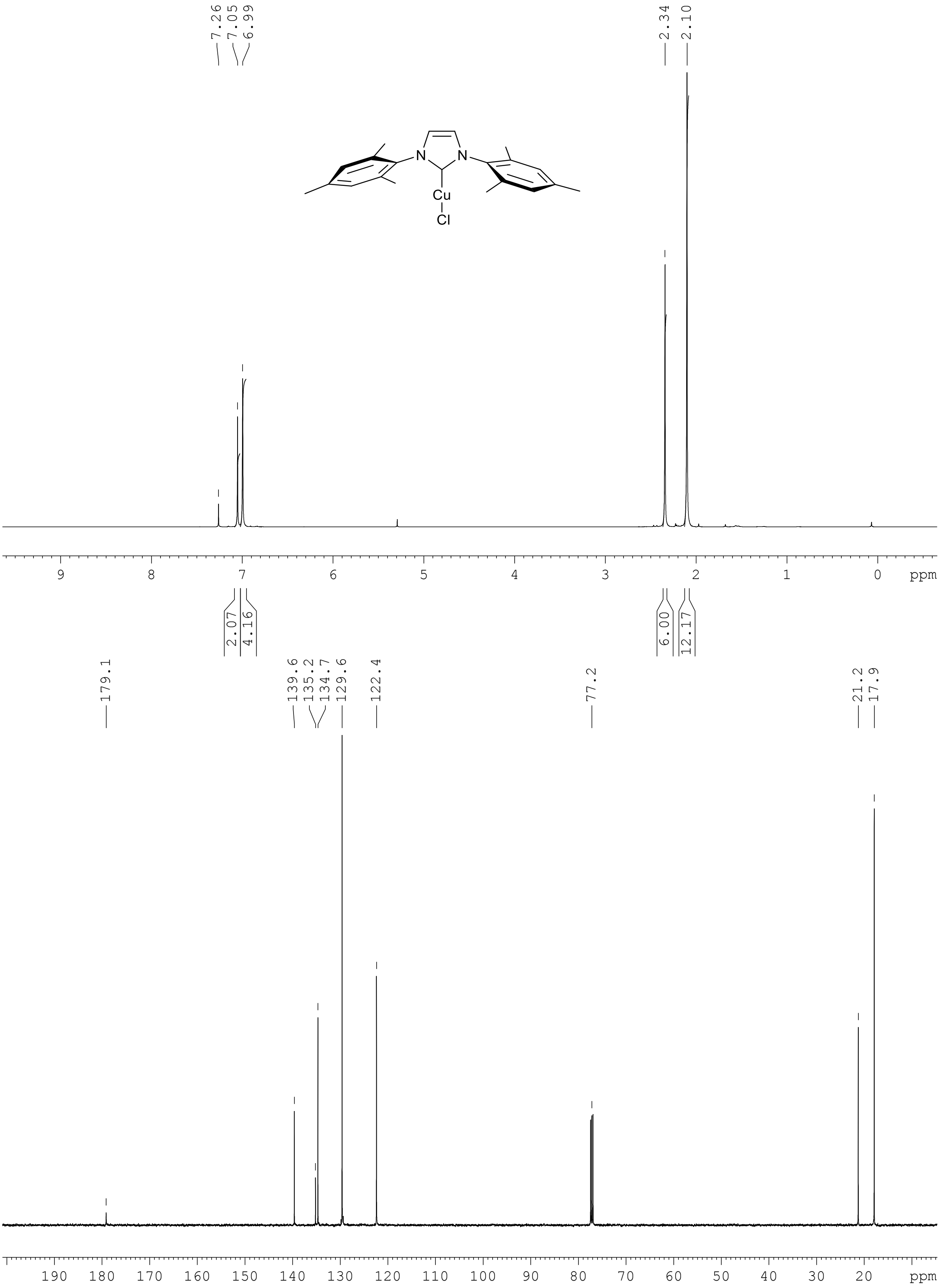

[Cu(Cl)(SIMes)] **2d**, ^{1}H NMR, $CDCl_3$, 298K and ^{13}C-{^{1}H} NMR, $CDCl_3$, 298 K

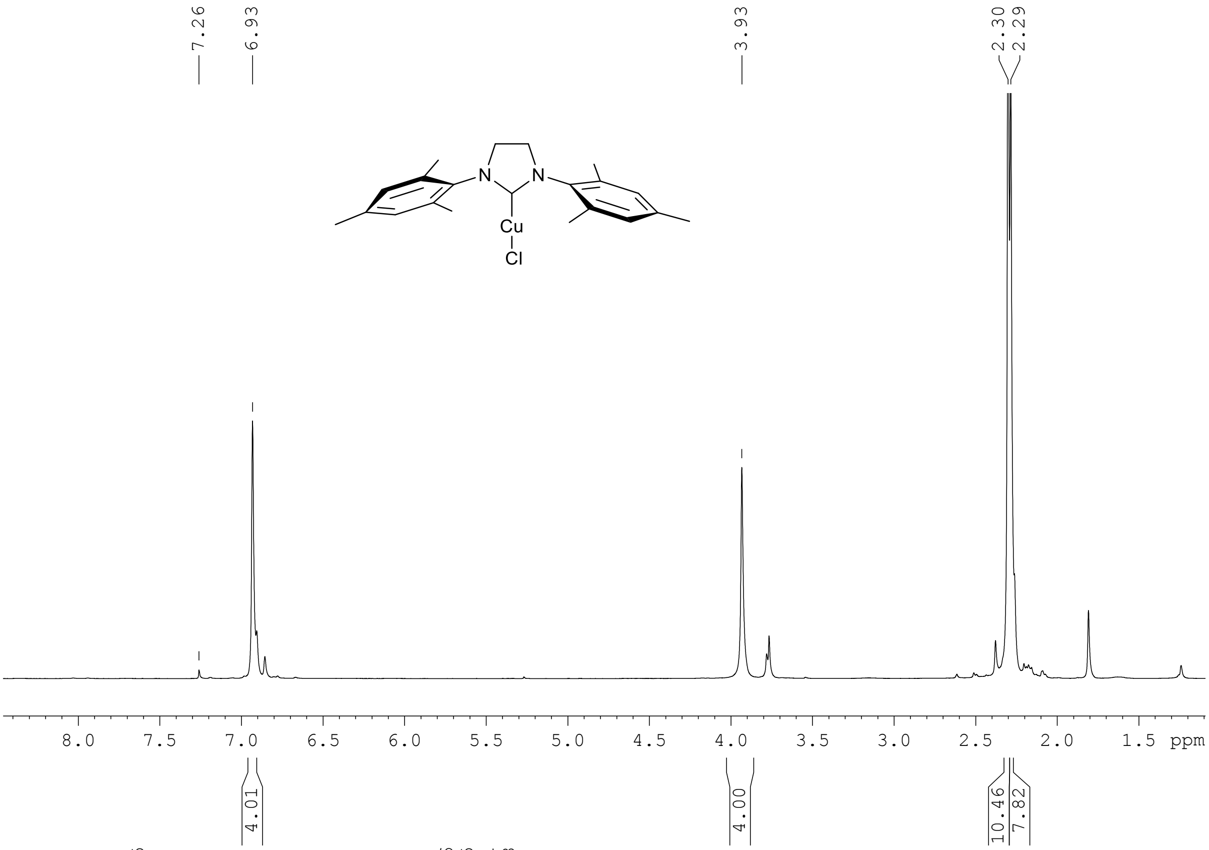

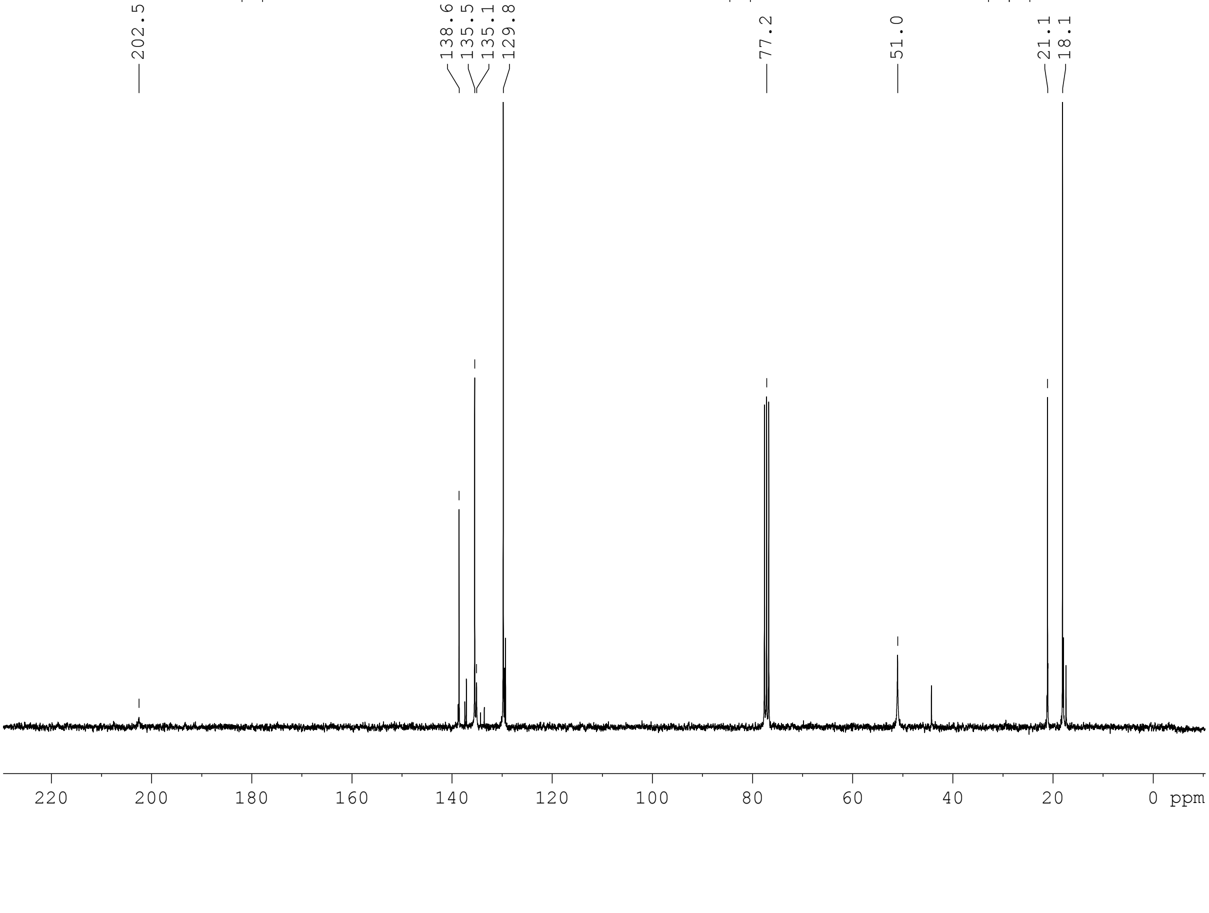

[Cu(Cl)(IPr*)] **2e**, ^{1}H NMR, $CDCl_3$, 298K and ^{13}C-{^{1}H} NMR, $CDCl_3$, 298 K

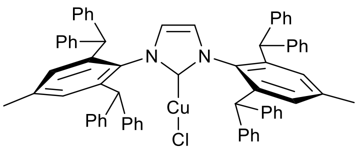

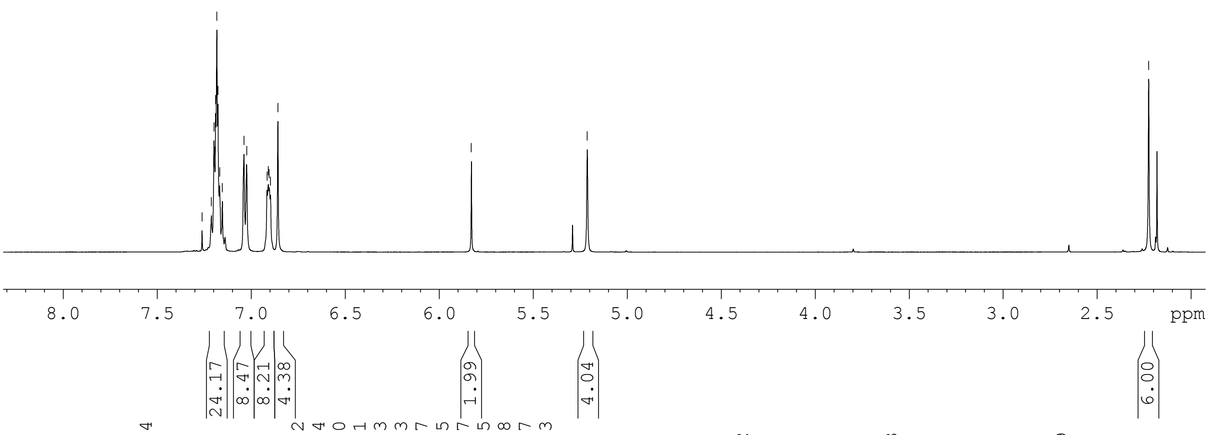

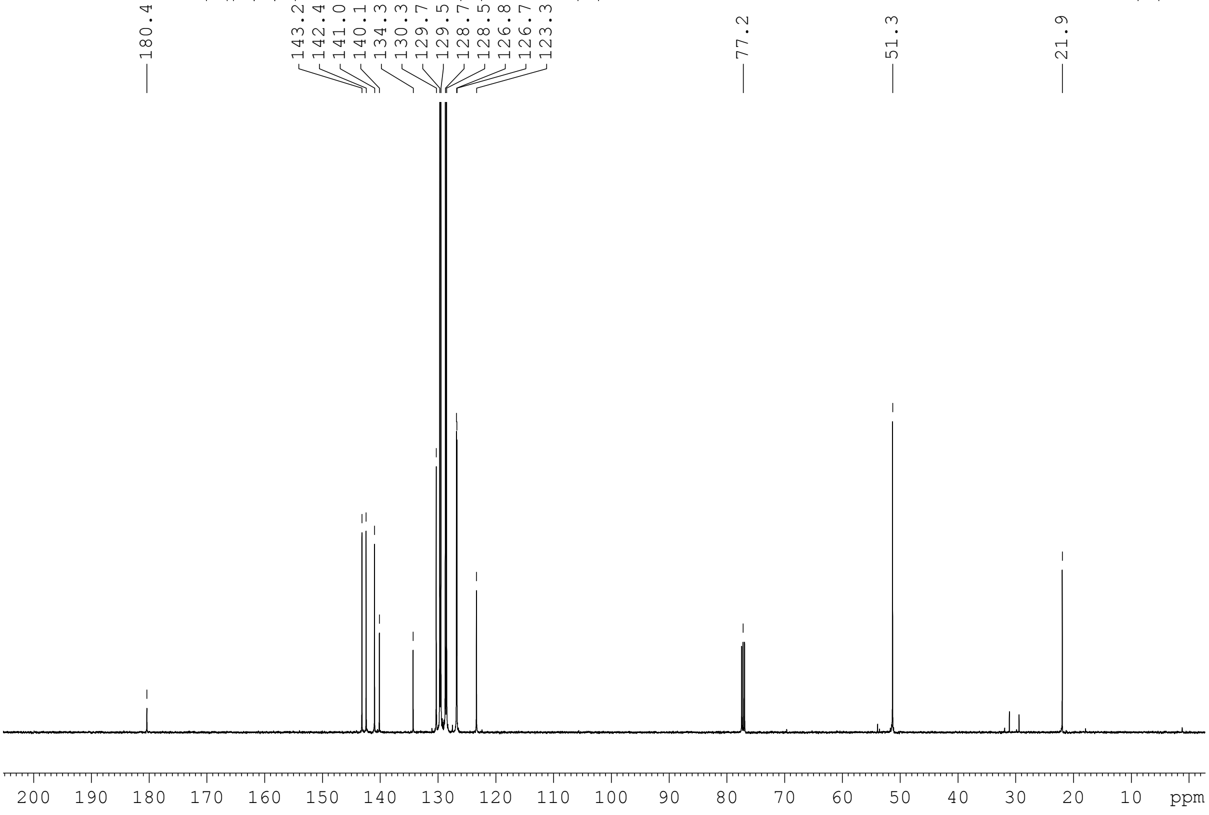

[Cu(Cl)(I^{t}Bu)] **2f**, ^{1}H NMR, $CDCl_3$, 298K and ^{13}C-{^{1}H} NMR, $CDCl_3$, 298 K

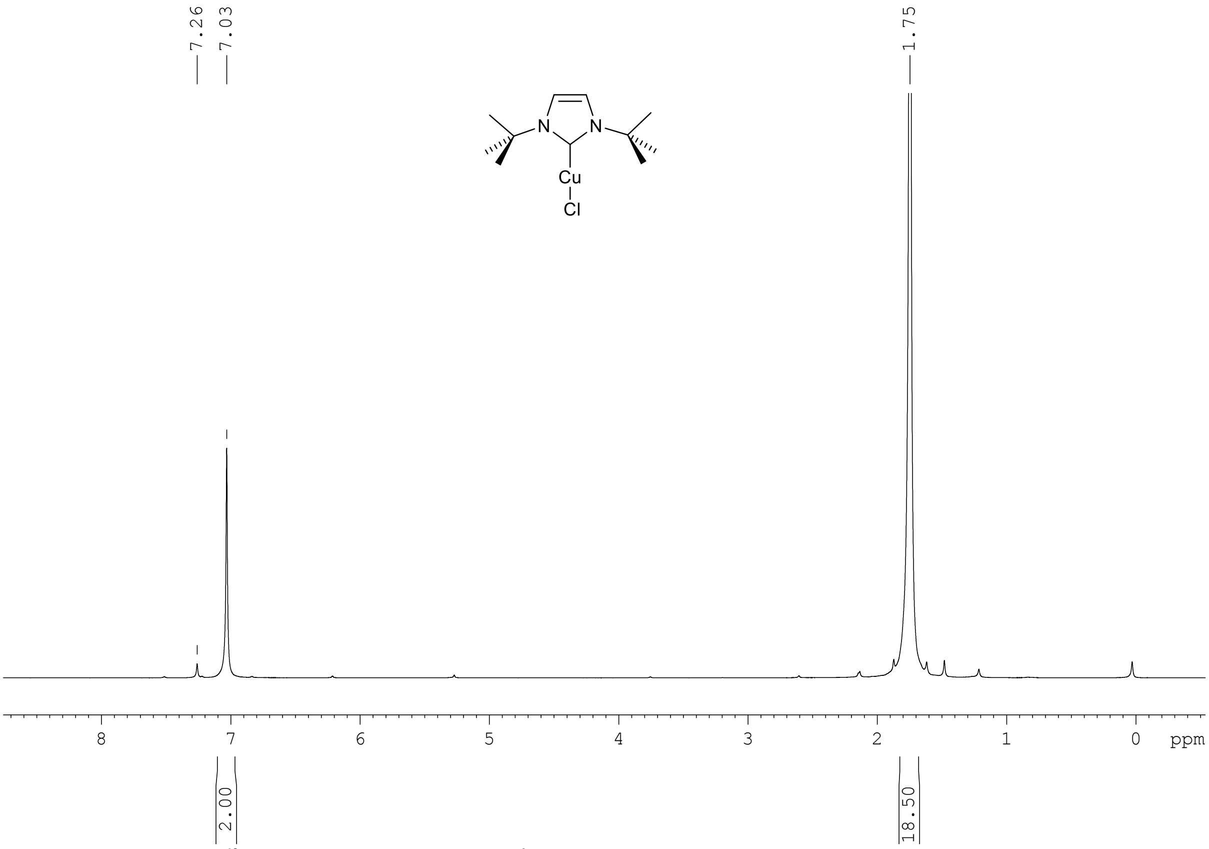

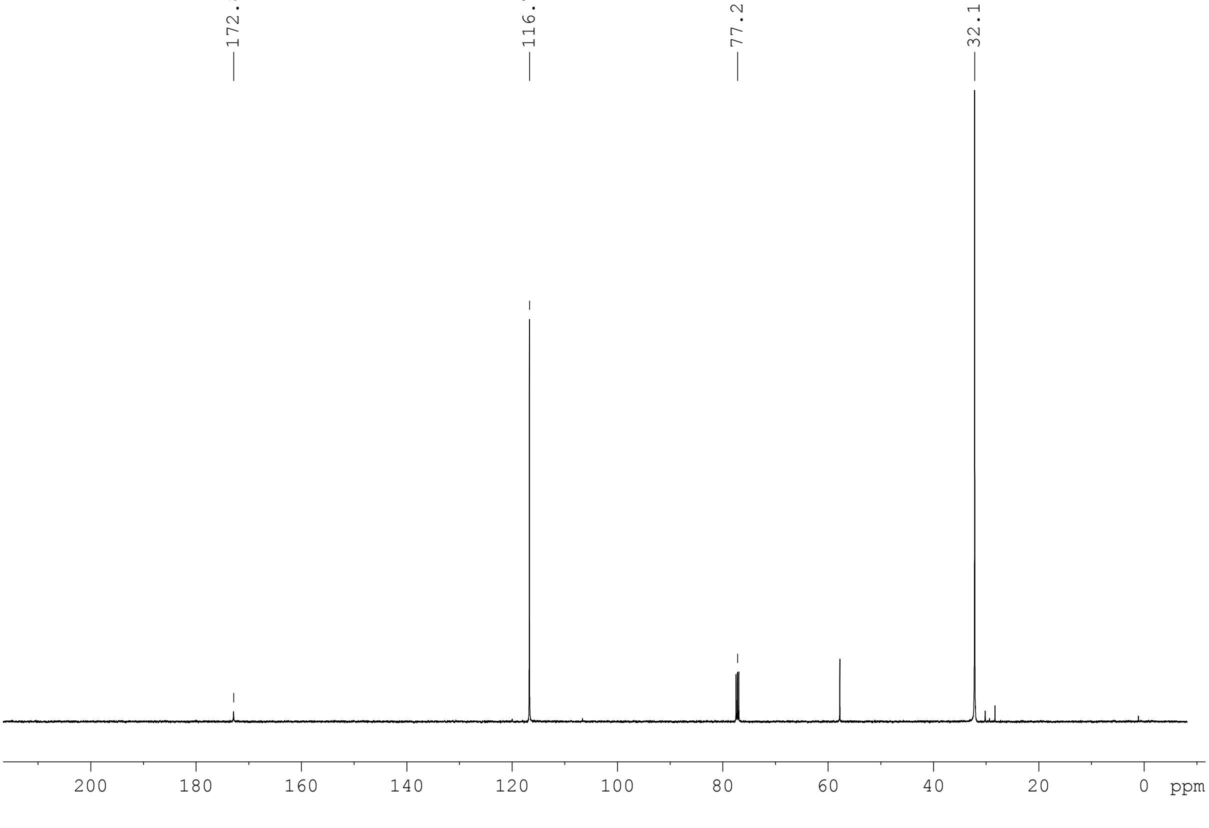

[Cu(Cl)(ICy)] **2g**, ^{1}H NMR, $CDCl_3$, 298K and ^{13}C-{^{1}H} NMR, $CDCl_3$, 298 K

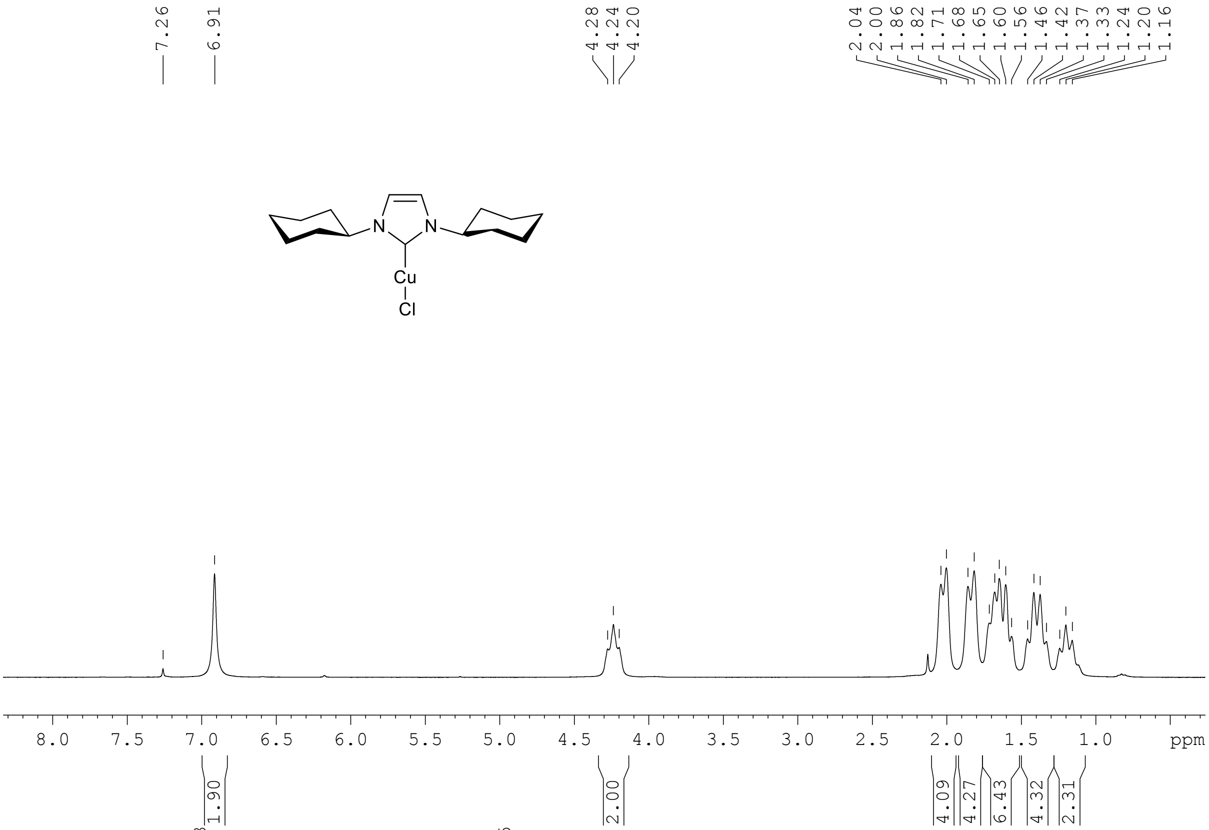

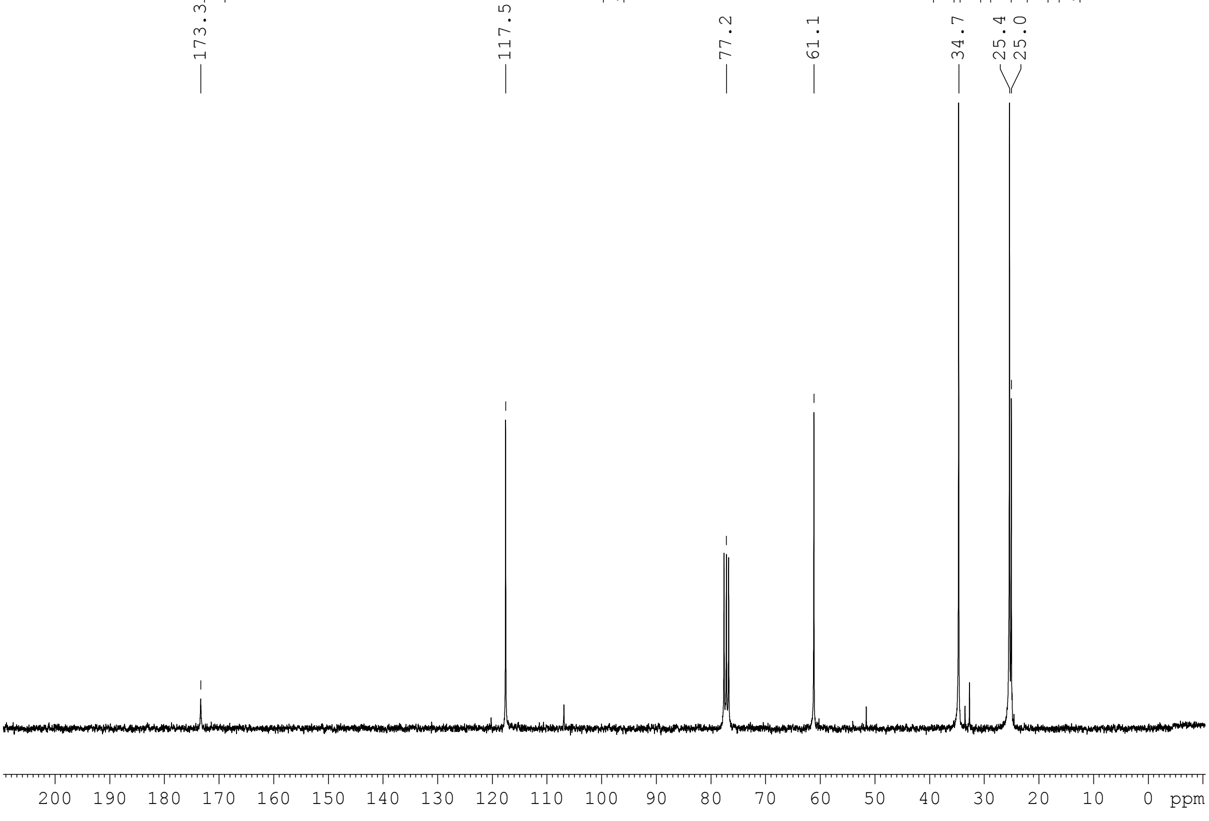

[Cu(Cl)(SICy)] **2h**, ^{1}H NMR, $CDCl_3$, 298K and ^{13}C-{^{1}H} NMR, $CDCl_3$, 298 K

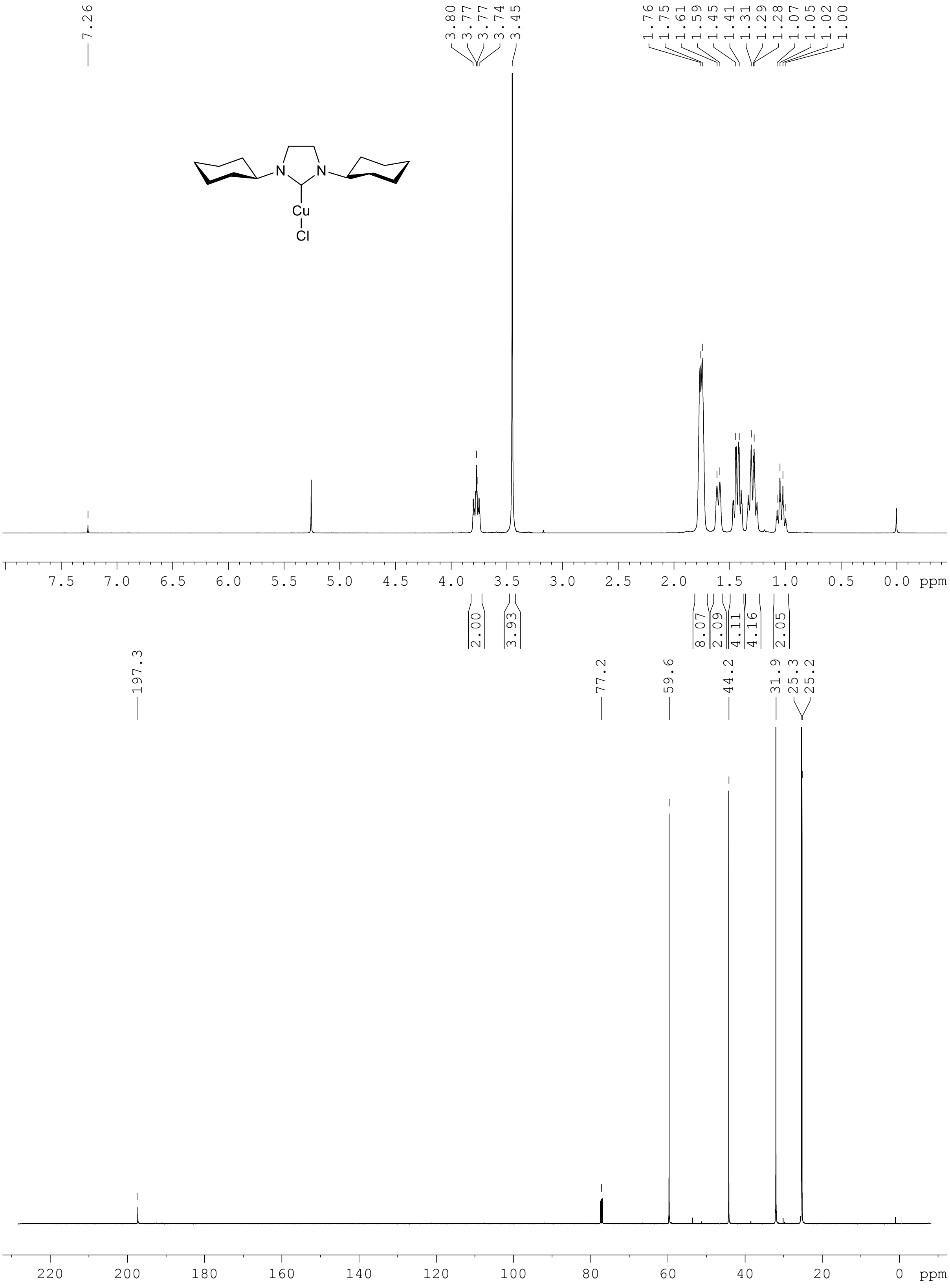

[Cu(Br)(IPr)] **2i**, ^{1}H NMR, $CDCl_3$, 298K.and ^{13}C-{^{1}H} NMR, $CDCl_3$, 298 K

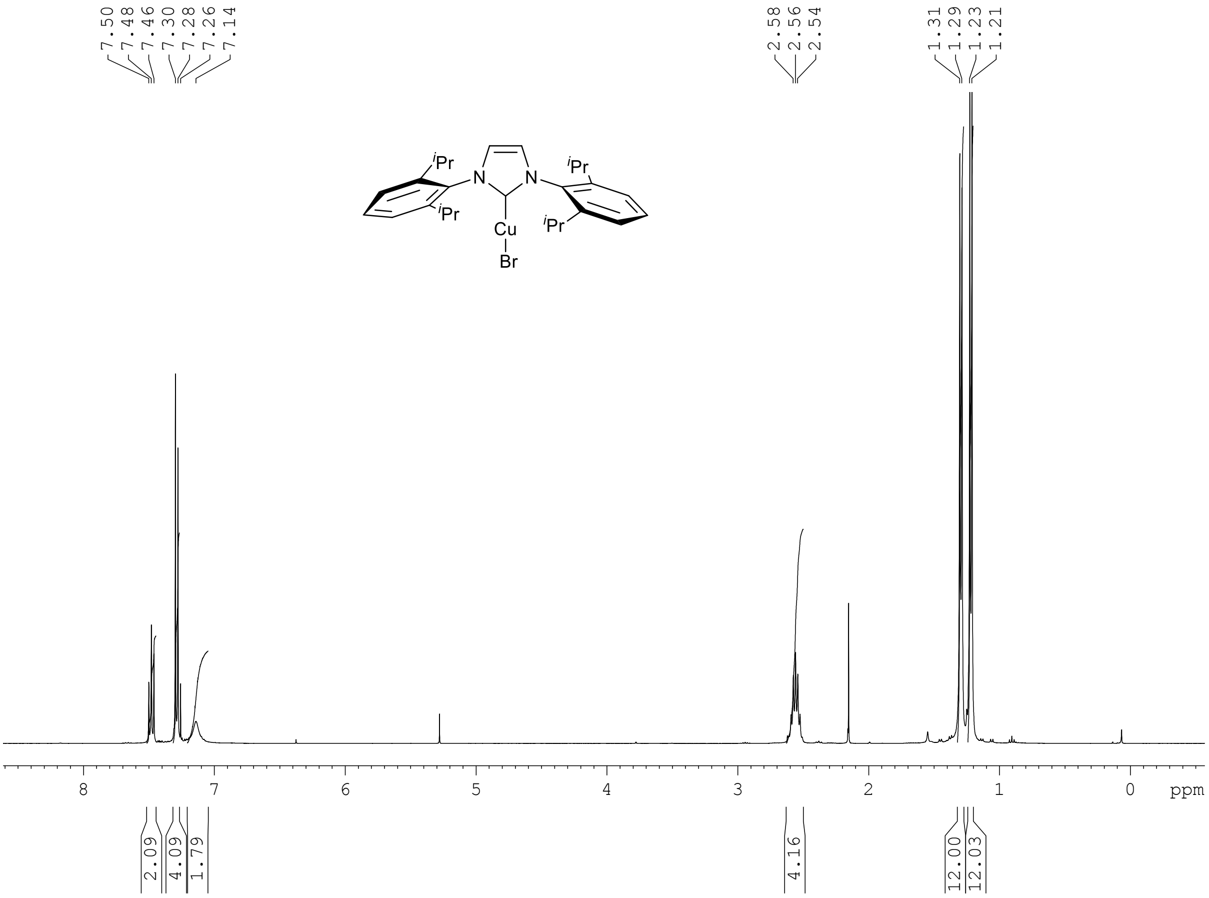

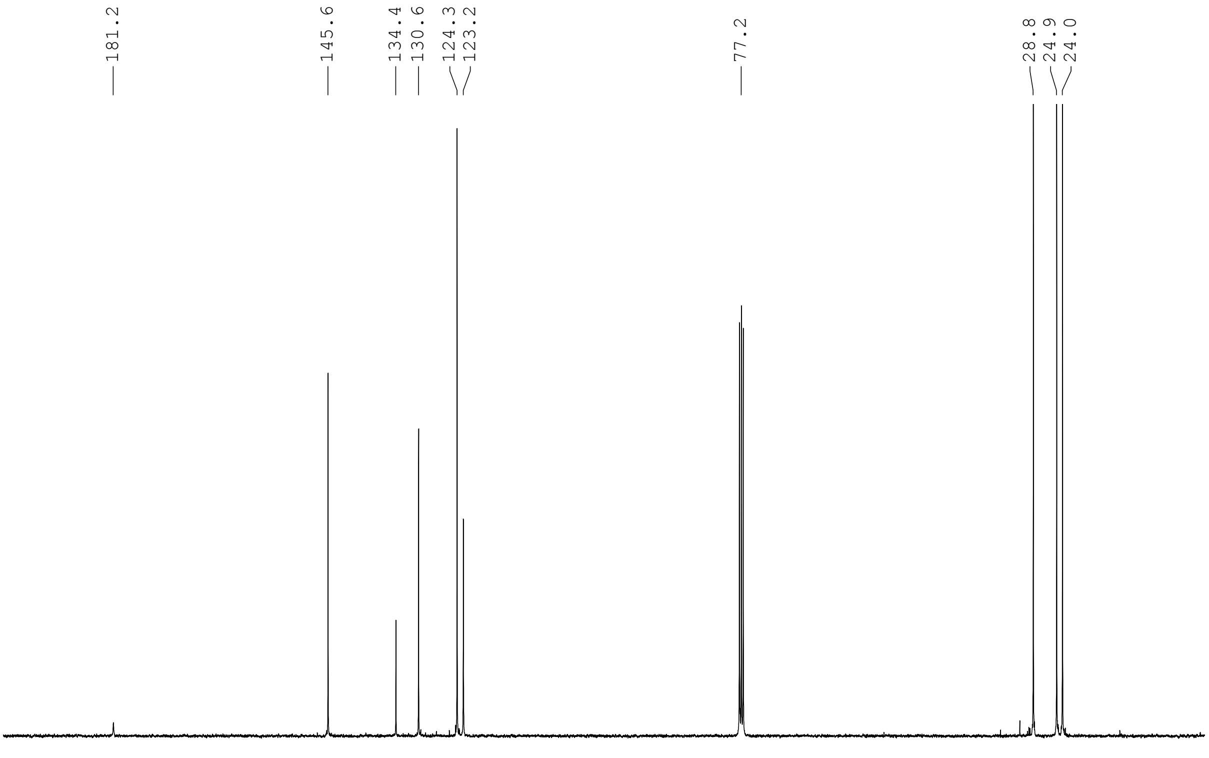

[Cu(I)(IPr)] **2j**, ^{1}H NMR, $CDCl_3$, 298K.and ^{13}C-{^{1}H} NMR, $CDCl_3$, 298 K

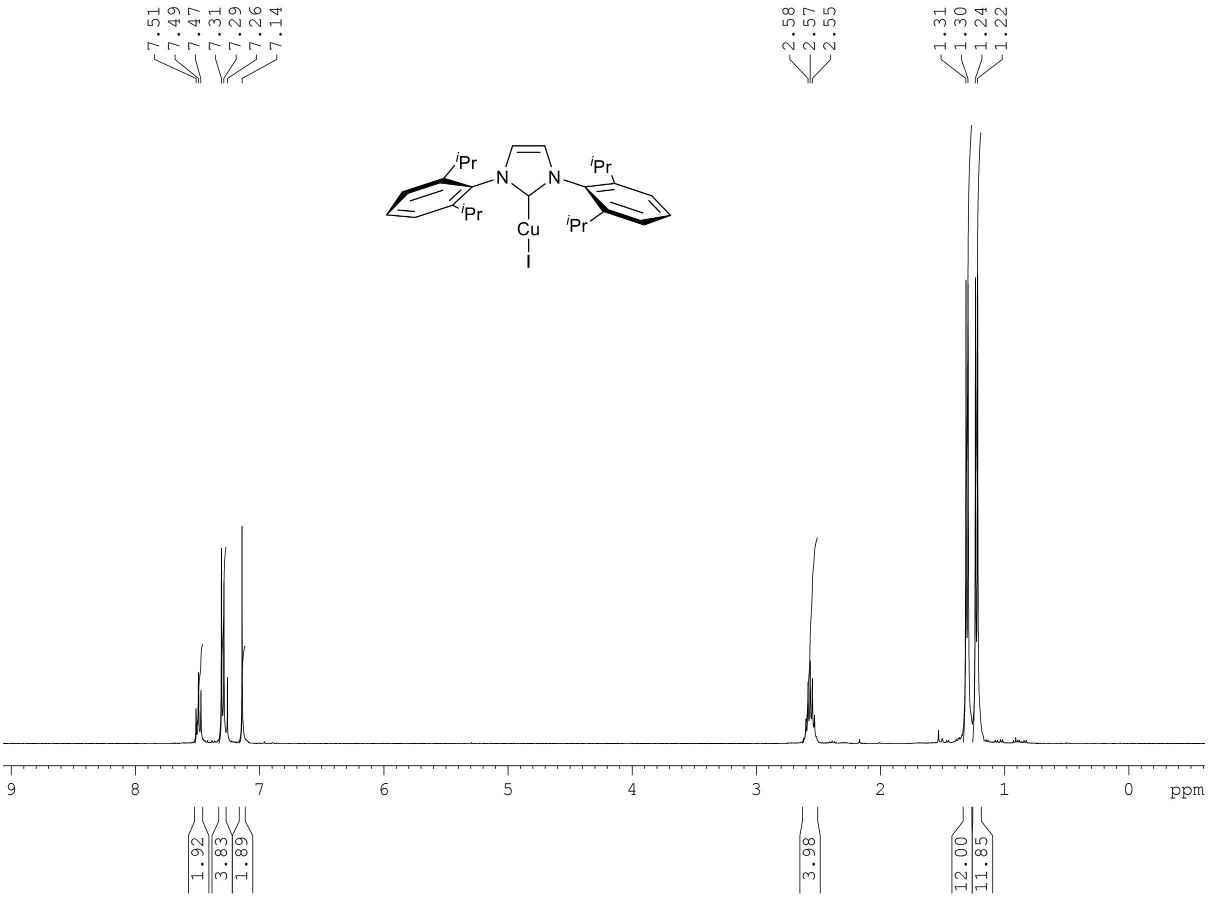

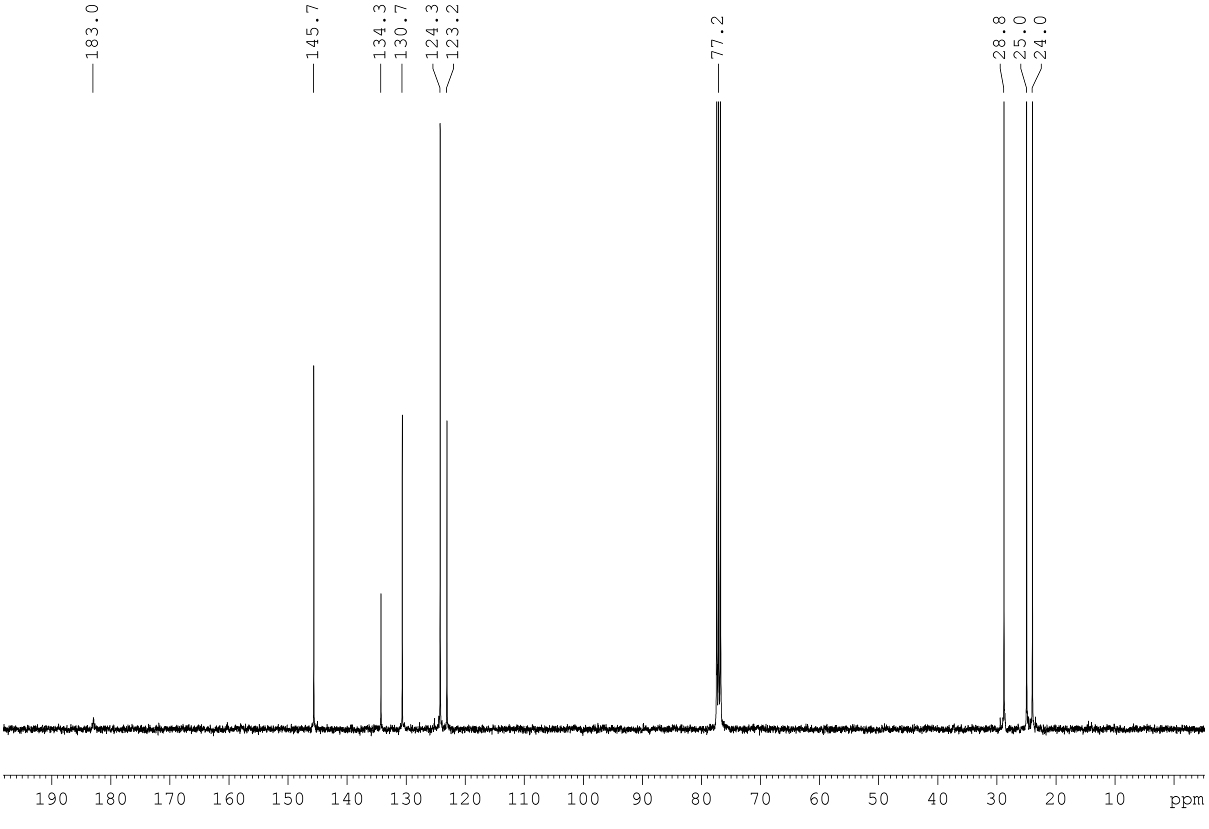

[IPrH][$CuCl_2$] **3a**, ^{1}H NMR, CD_2Cl_2, 298K.and $^{13}C-\{^{1}H\}$ NMR, CD_2Cl_2, 298 K

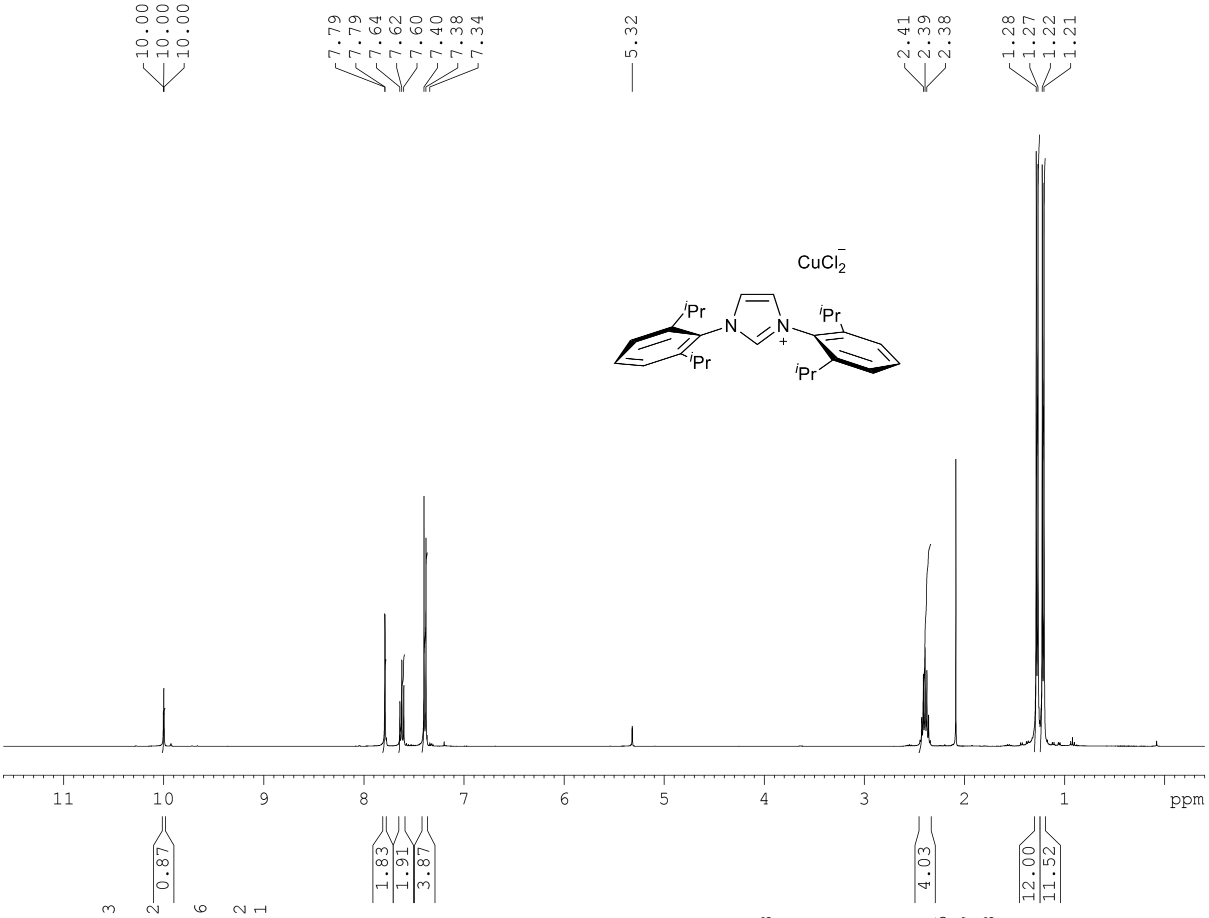

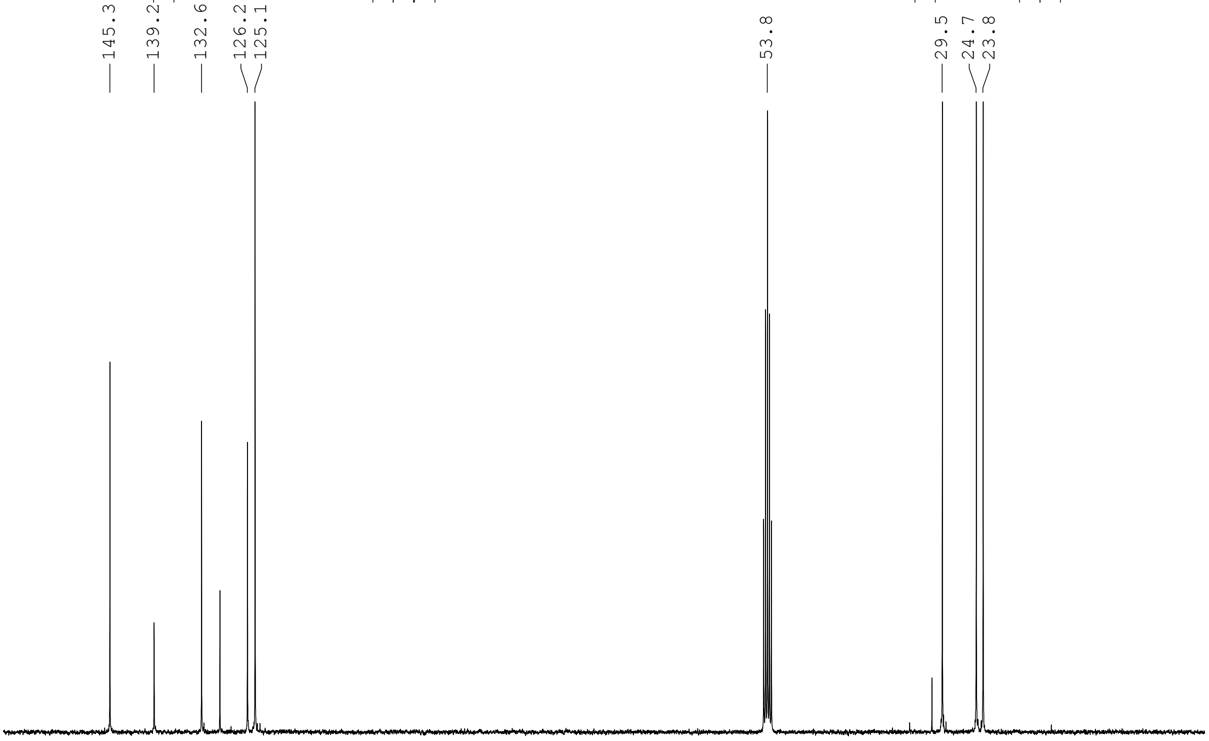

[SIPrH][CuCl$_2$] **3b**, ^{1}H NMR, CD_2Cl_2, 298K.and ^{13}C-{^{1}H} NMR, CD_2Cl_2, 298 K

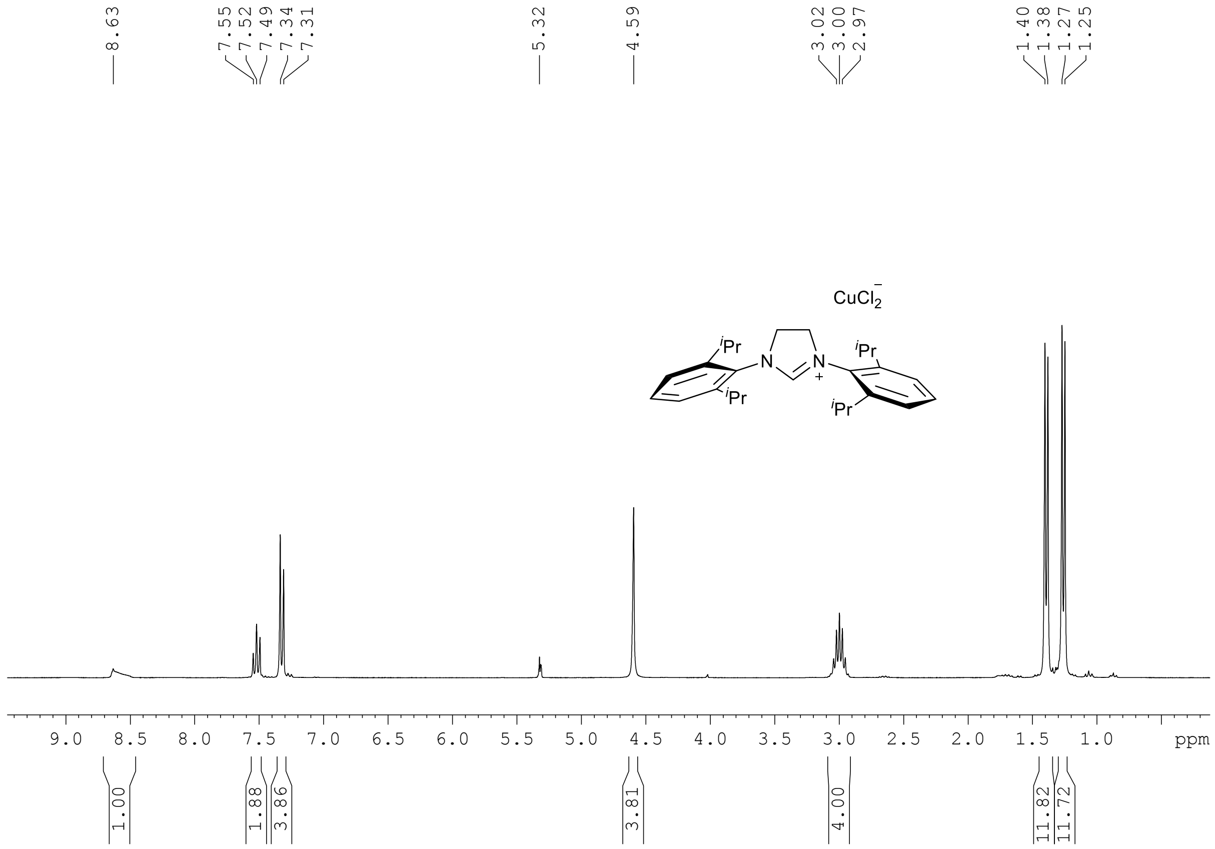

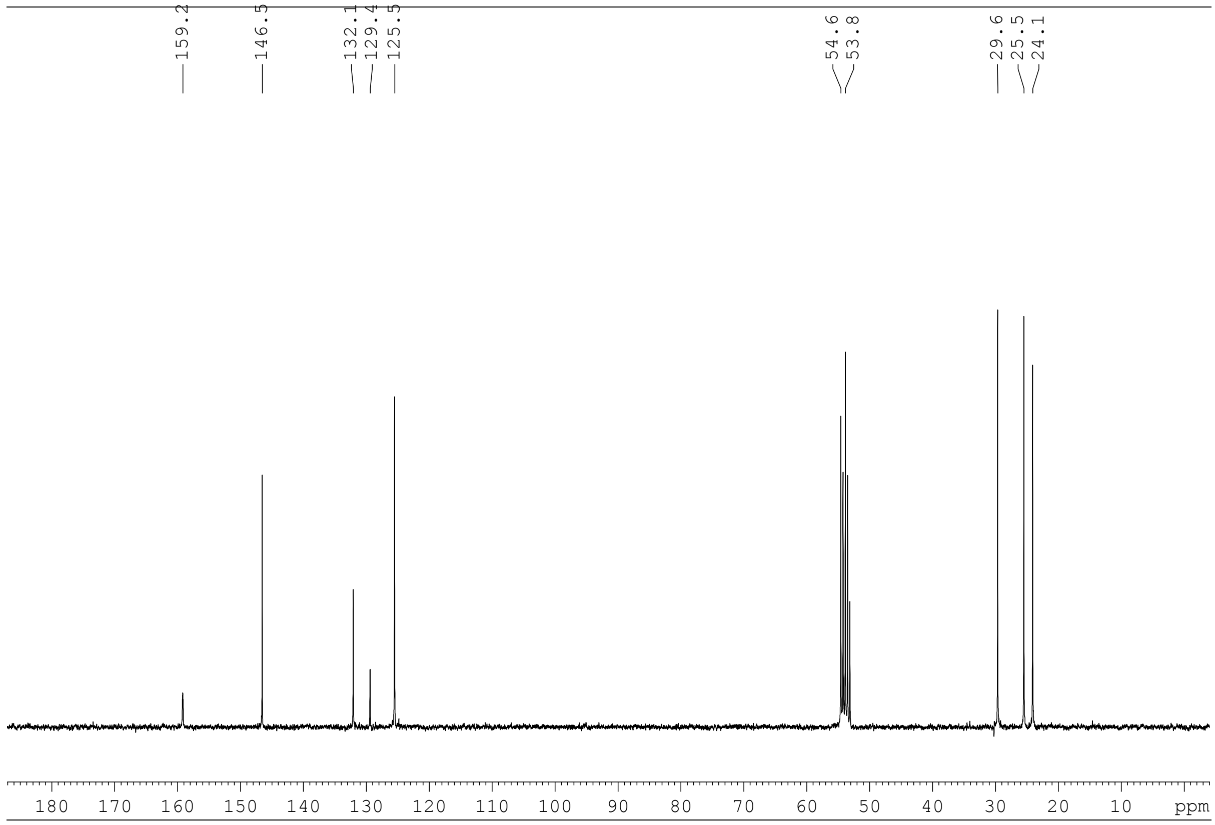

[IMesH][$CuCl_2$] **3c**, ^1H NMR, CD_2Cl_2, 298K.and ^{13}C-{^1H} NMR, CD_2Cl_2, 298 K

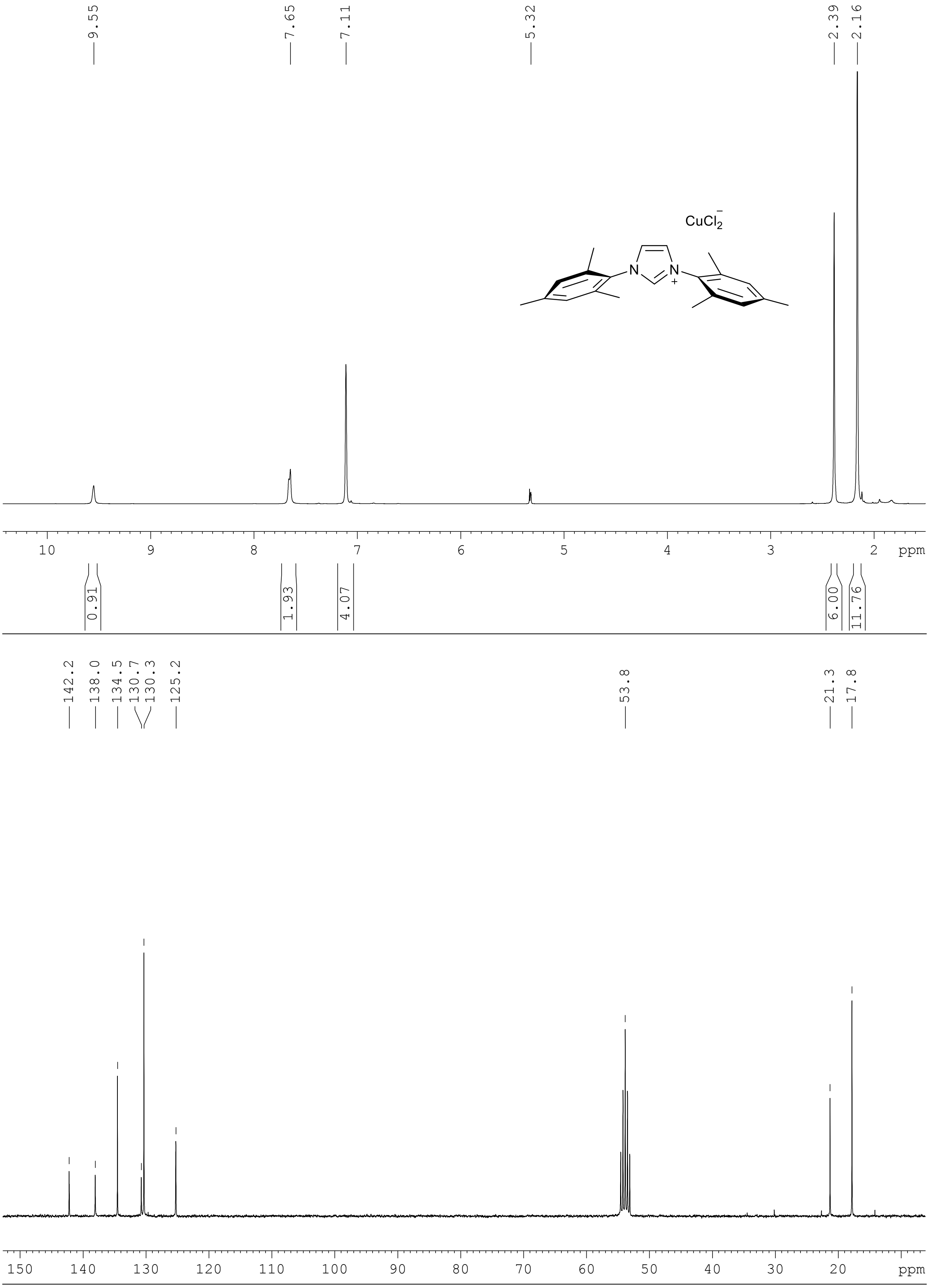

[SIMesH][CuCl$_2$] **3d**, ^{1}H NMR, CD$_2$Cl$_2$, 298K.and ^{13}C-{^{1}H} NMR, CD$_2$Cl$_2$, 298 K

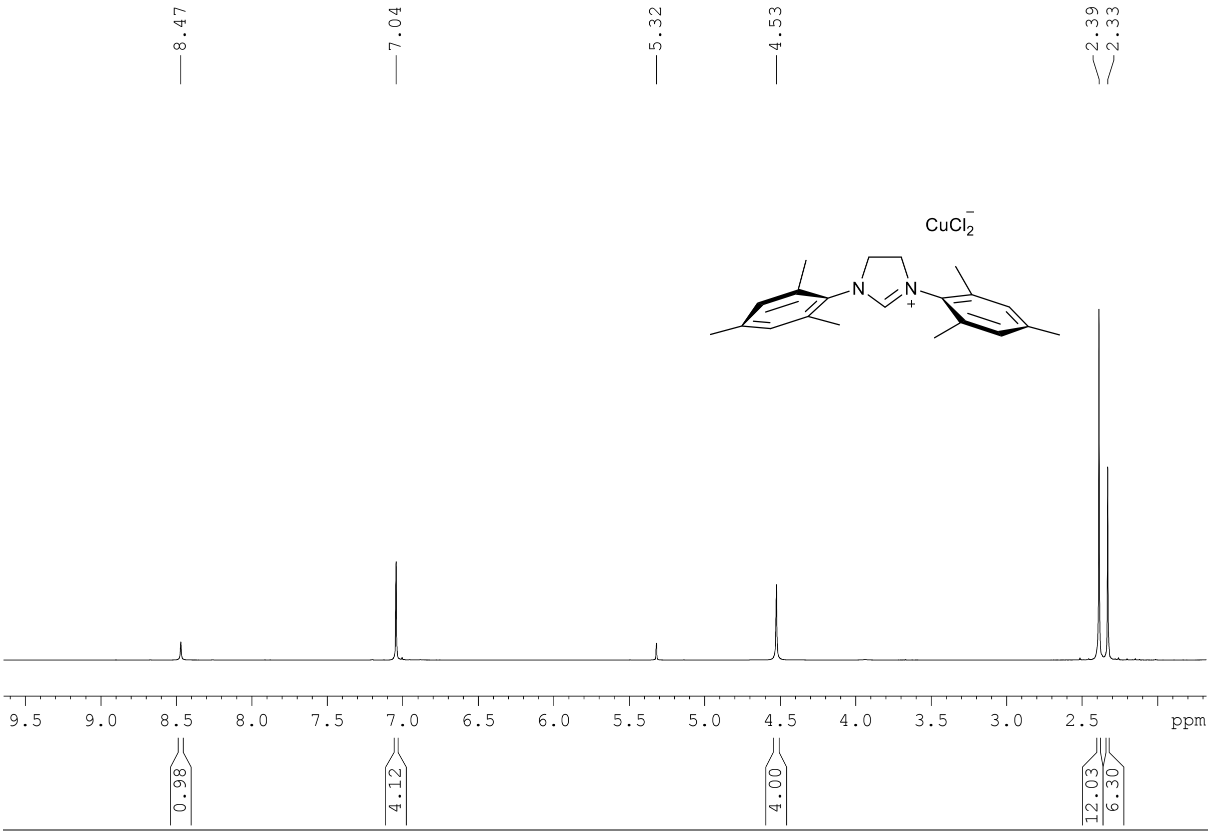

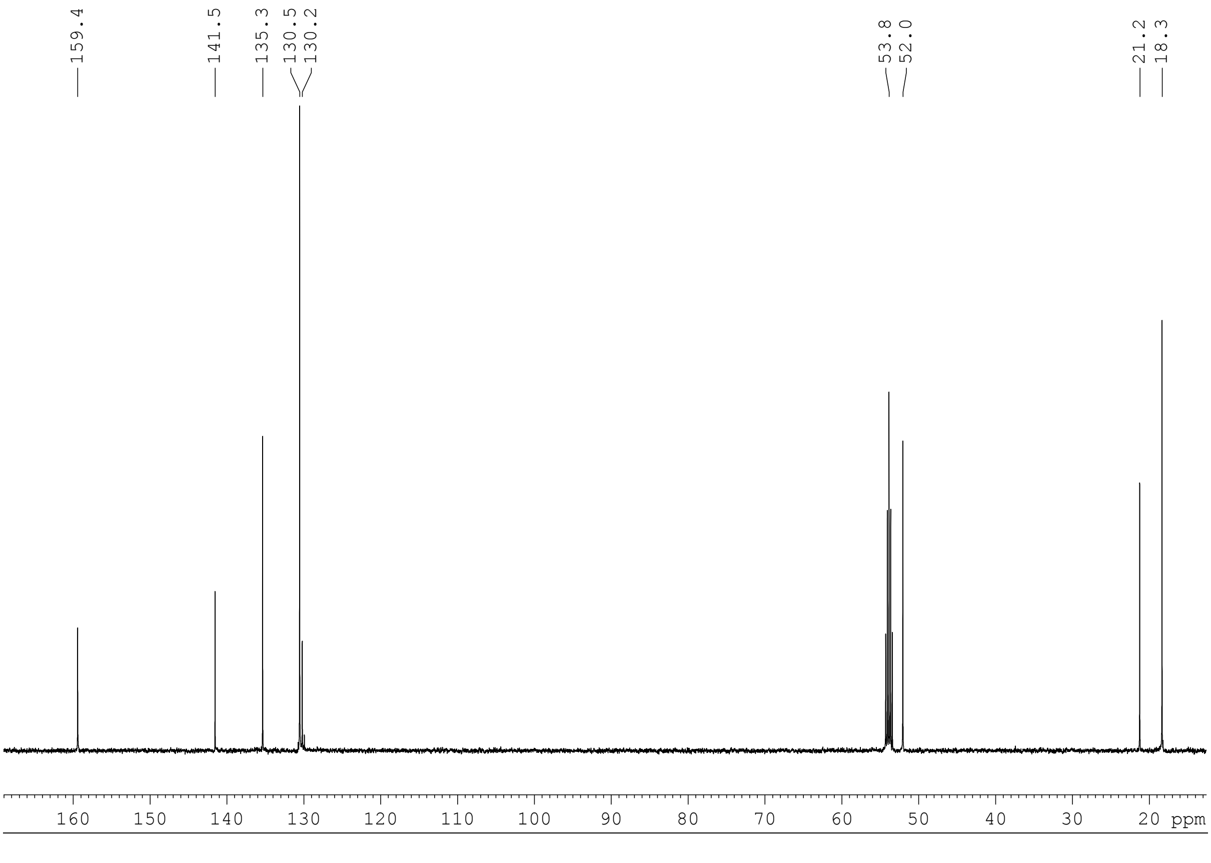

[ICyH][$CuCl_2$] **3g**, ^{1}H NMR, $CDCl_3$, 298K.and ^{13}C-{^{1}H} NMR, $CDCl_3$, 298 K

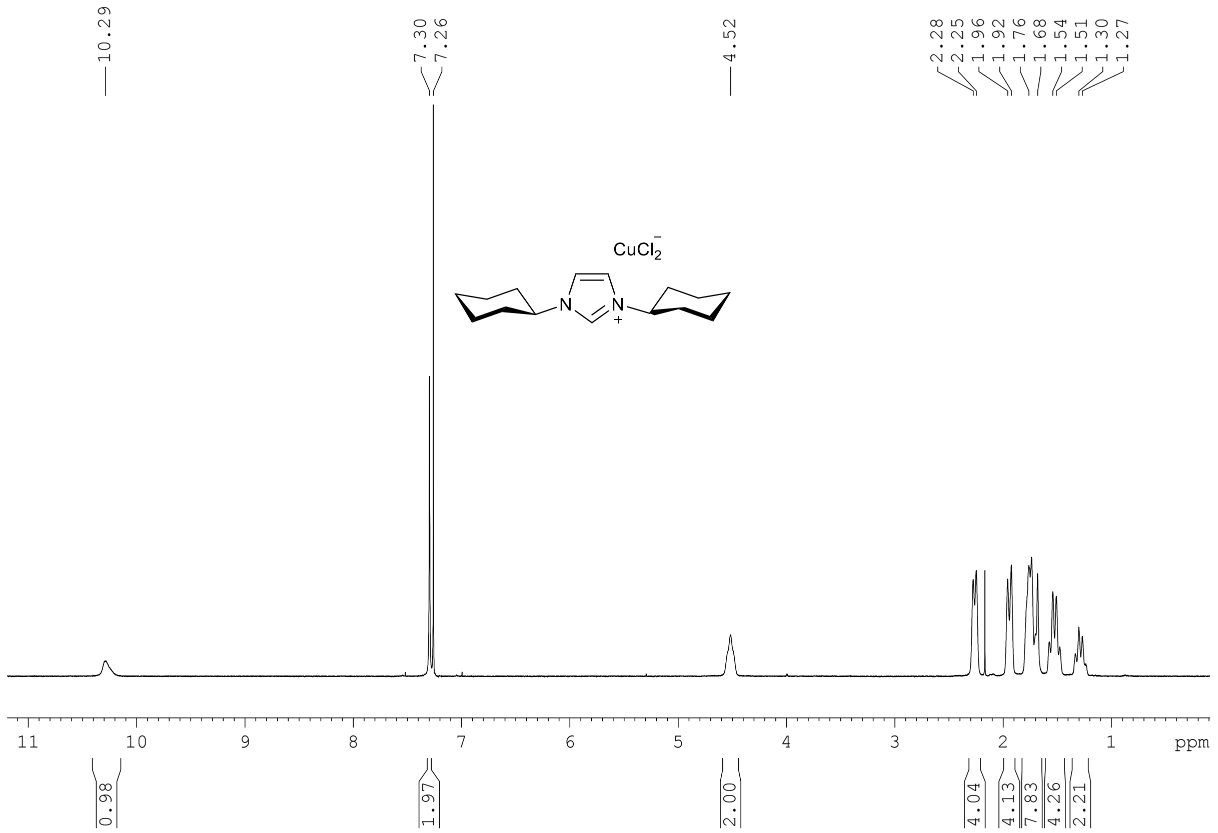

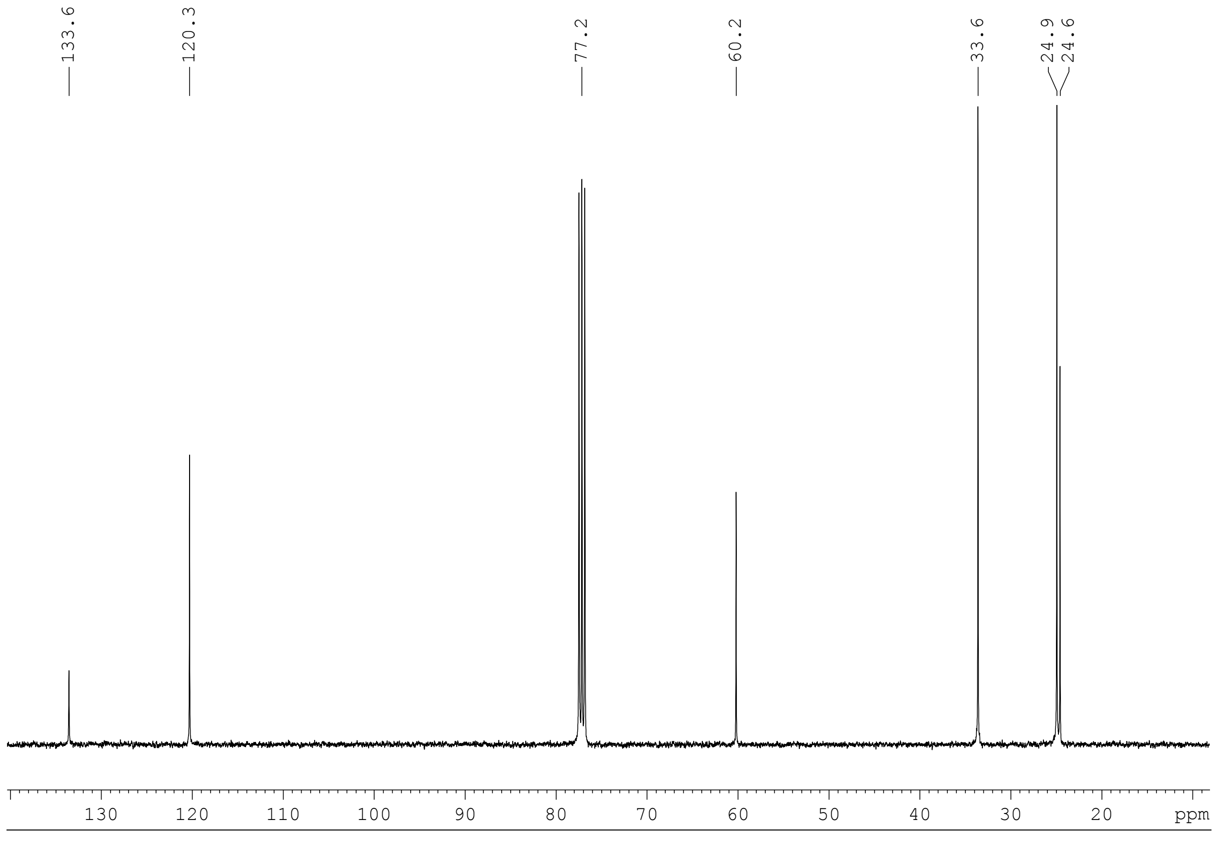

[IPrH][CuClBr] **3i**, ^{1}H NMR, CD_2Cl_2, 298K.and ^{13}C-{^{1}H} NMR, CD_2Cl_2, 298 K

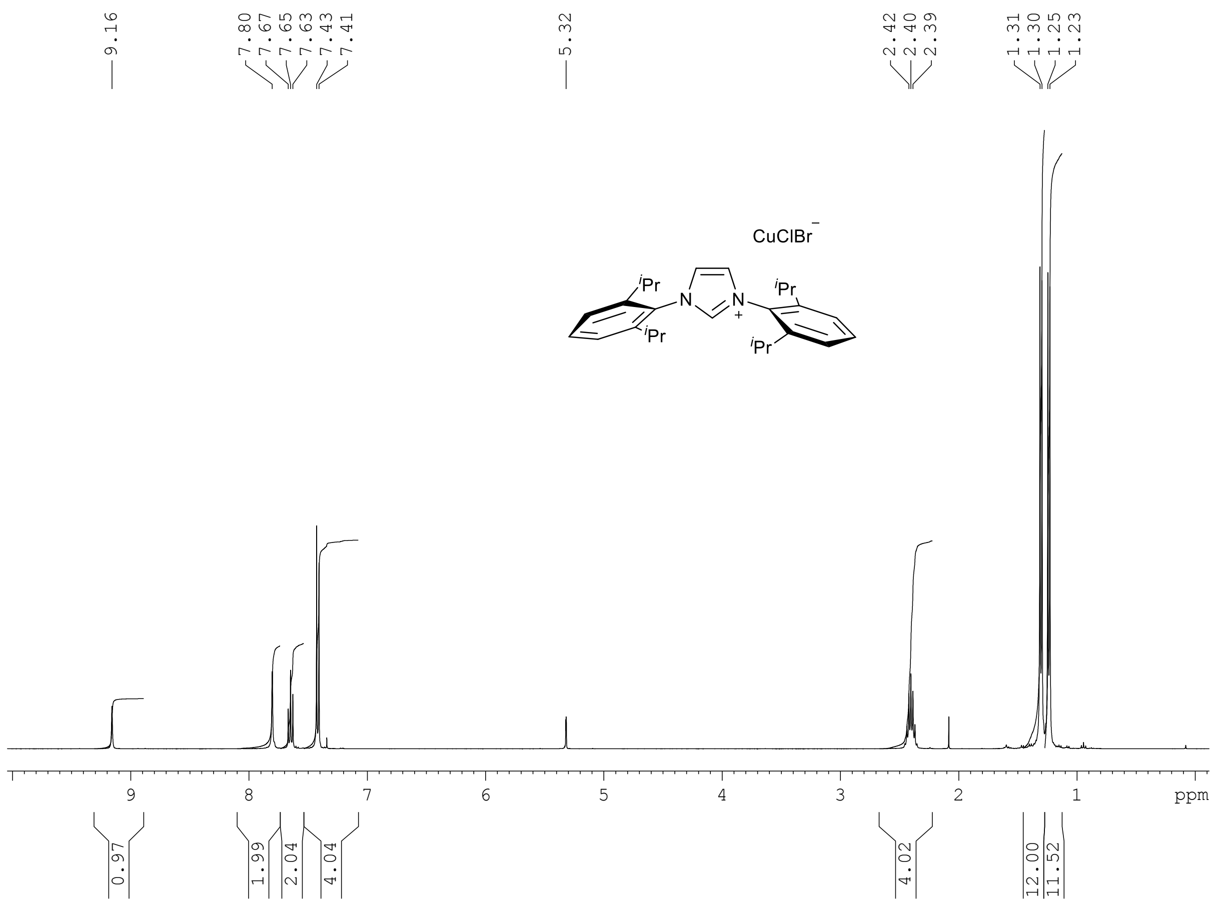

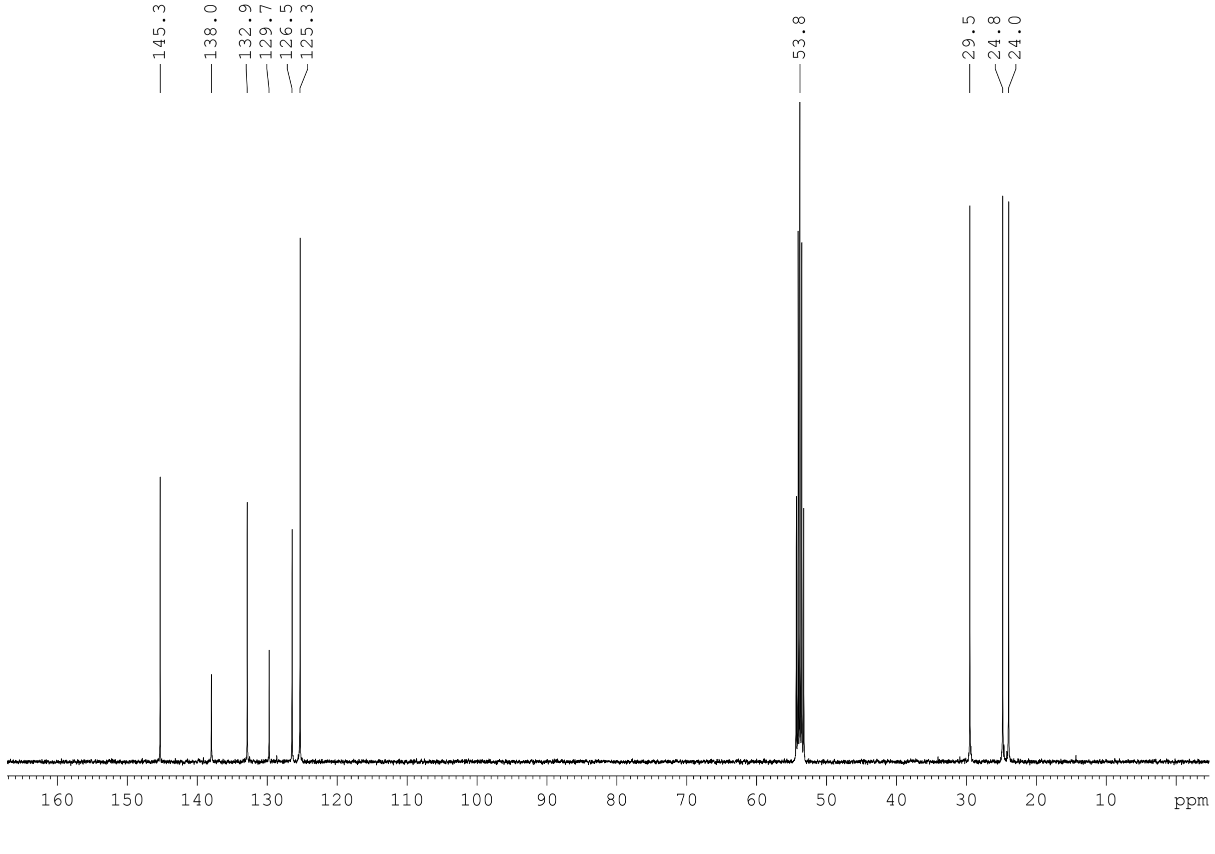

[IPrH][CuClI] **3j**, ^{1}H NMR, CD_2Cl_2, 298K.and ^{13}C-{^{1}H} NMR, CD_2Cl_2, 298 K

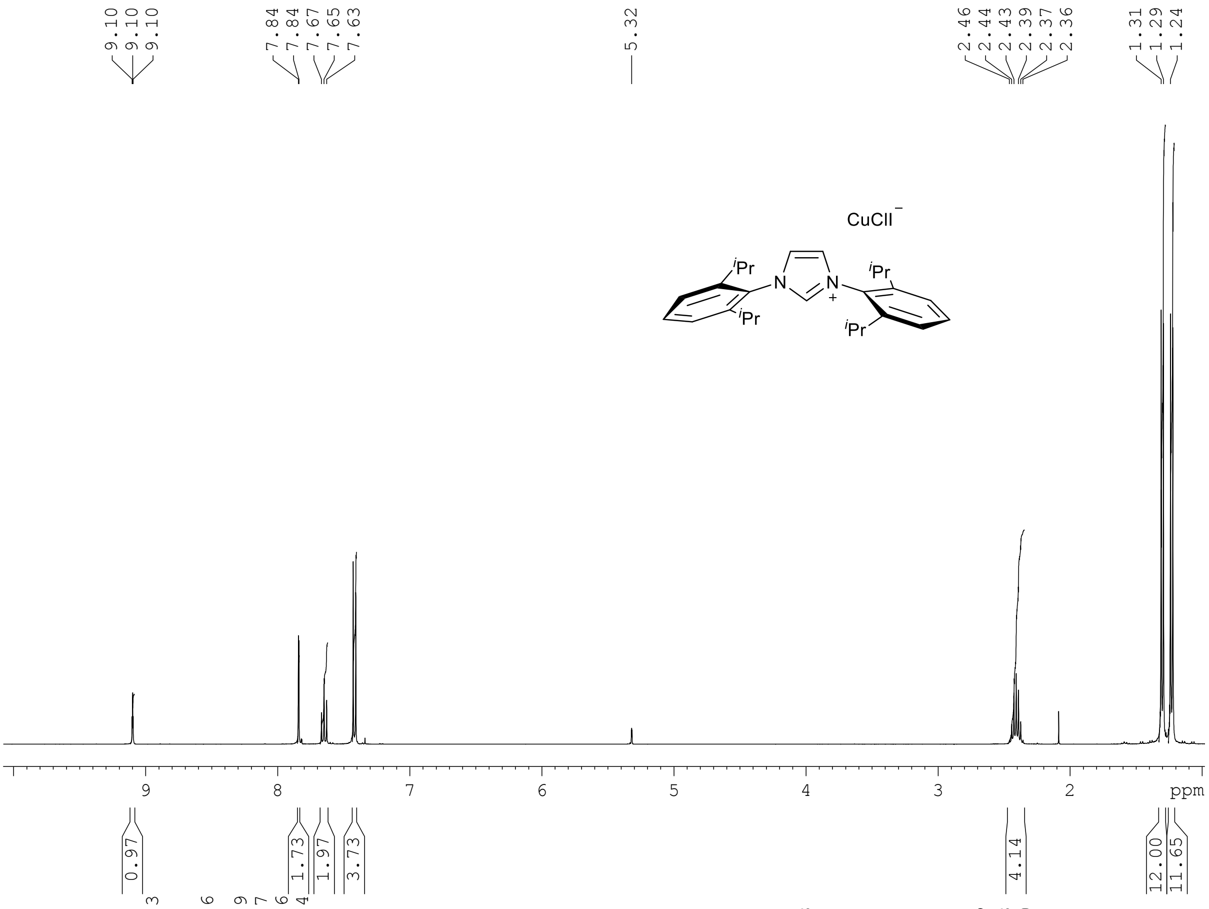

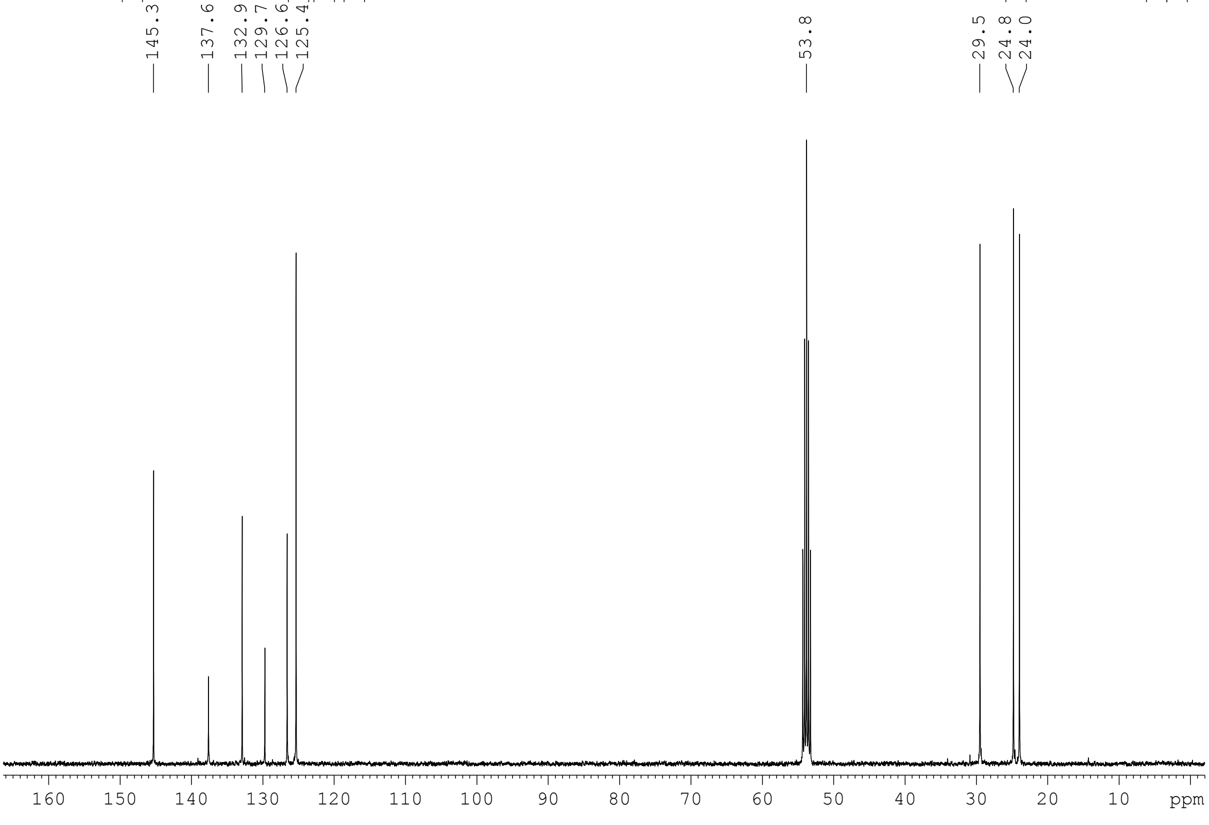

[IPrH][CuBrI] **3k**, ^{1}H NMR, CD_2Cl_2, 298K.and ^{13}C-{^{1}H} NMR, CD_2Cl_2, 298 K

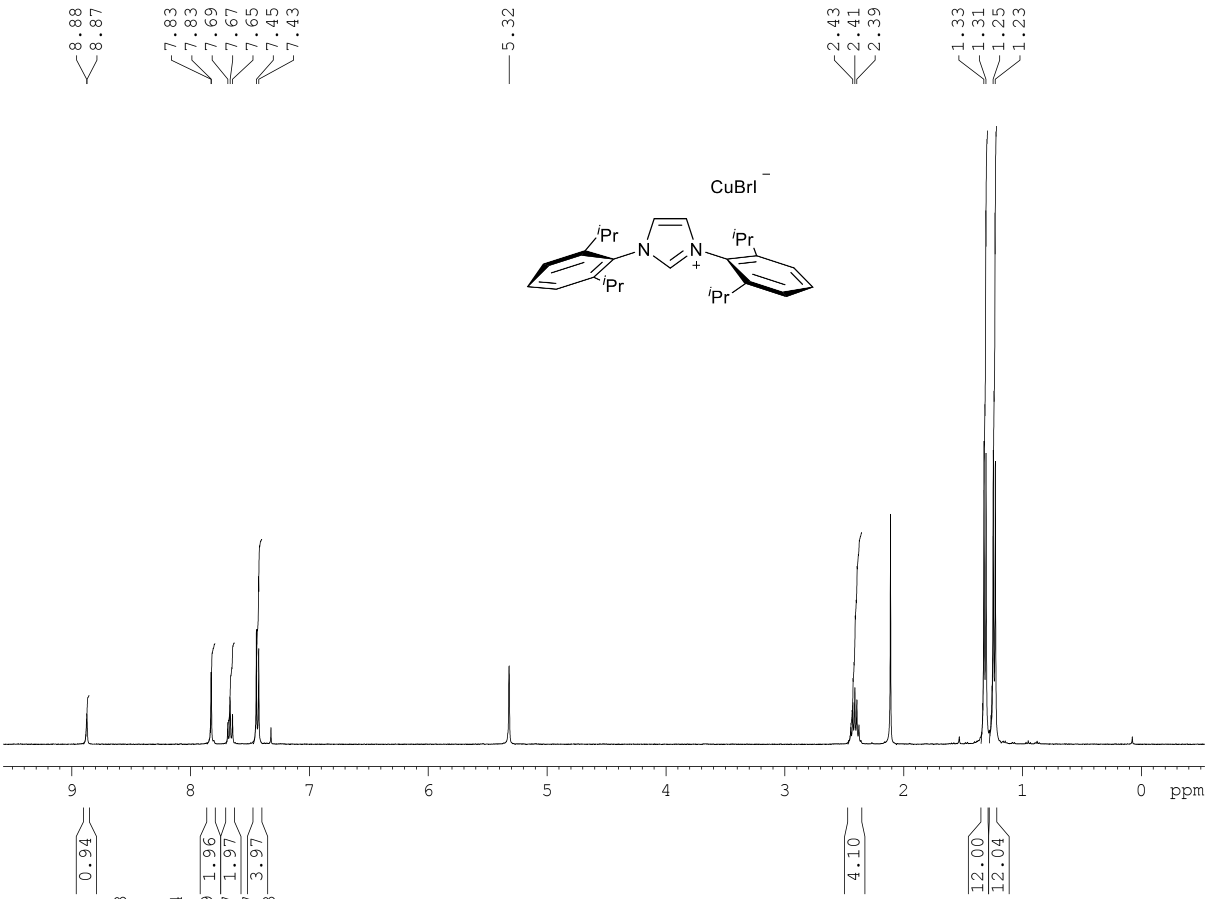

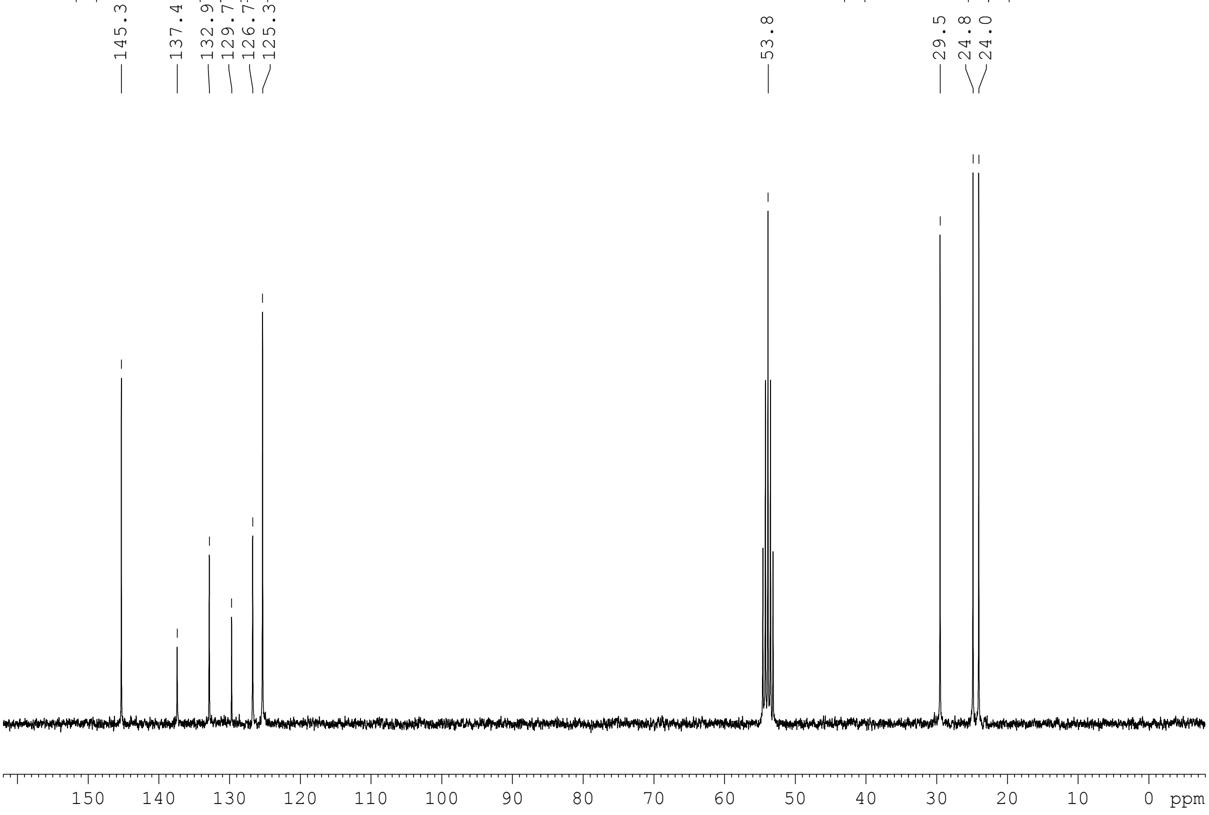

Crystal data and Structure refinement

[SIPrH][$CuCl_2$]/CCDC-940850 (**3b**)

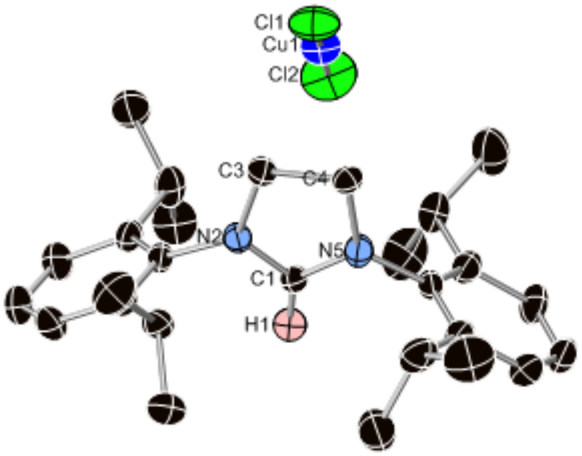

[IMesH][$CuCl_2$]/CCDC-940851 (**3c**)

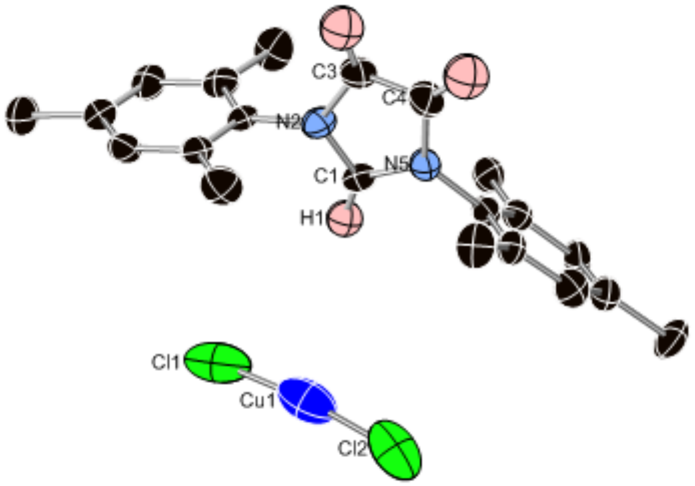

[SIMes][$CuCl_2$]/CCDC-940852 (**3d**)

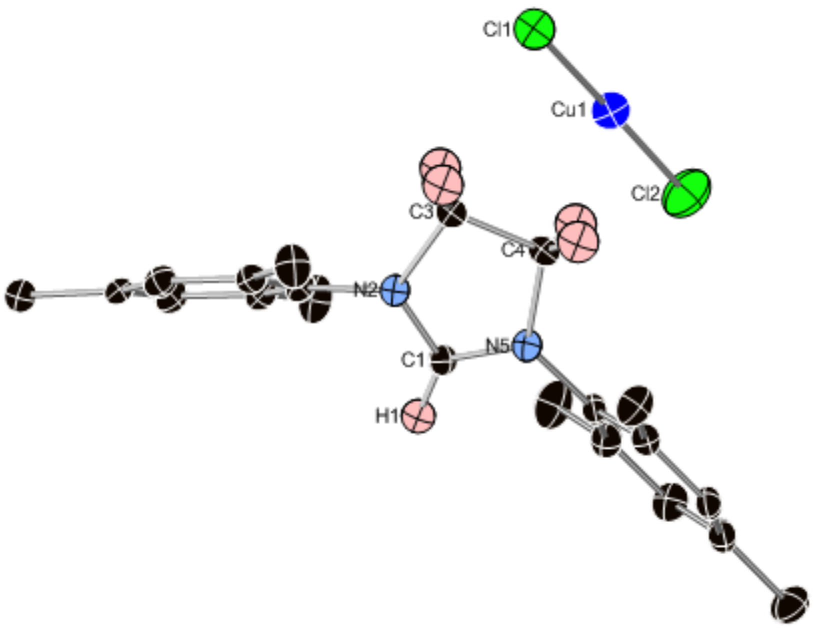

[SIMes][$CuCl_2$]/CCDC-940853 (**3g**)

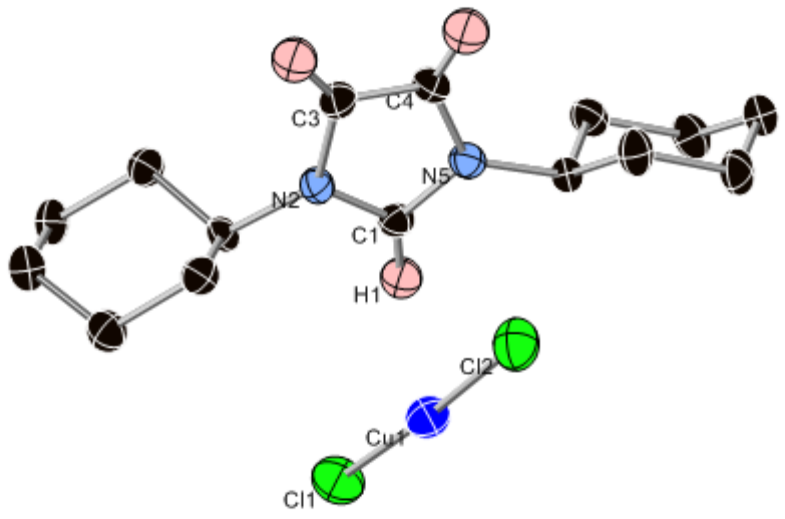

	CCDC/940850 **3b**	CCDC/940851 **3c**	CCDC/940852 **3d**	CCDC-940853 **3g**
Empirical formula	$C_{27}H_{39}Cl_2CuN_2$	$C_{21}H_{25}Cl_2CuN_2$	$C_{21}H_{27}Cl_2CuN_2$	$C_{15}H_{25}Cl_2CuN_2$
Formula weight	526.07	439.89	441.91	367.83
Temperature (K)	93	93	93	93
Wavelength (Å)	0.71075	0.71075	0.71075	0.71075
Crystal system	orthorhombic	monoclinic	monoclinic	monoclinic
Space group	$Pca2_1$	$P2_1/c$	$P2_1/c$	C2/c
a (Å)	14.746(4)	8.572(4)	8.388(3)	23.087(12)
b (Å)	19.910(4)	15.885 (7)	16.503(5)	9.877(4)
c (Å)	19.522(4)	15.852 (6)	15.847(5)	c 16.619(8)
α, β, γ (°)	90, 90, 90	90, 100.145(13), 90	90, 100.157(7), 90	90, 114.446(11), 90
Volume $(Å)^3$	5731(2)	2124.9(15)	2159.2(11)	3450(3)
Z	8	4	4	8
Density calculated (g/cm^3)	1.219	1.375	1.359	1.416
Absorption coefficient (mm^{-1})	0.96	1.286	1.266	1.568
F(000)	2224.00	912.00	920.00	1536.00
Crystal size (mm^3)	0.20 x 0.20 x 0.08	0.10 × 0.10 × 0.05	0.12 × 0.12 × 0.12	0.12 × 0.12 × 0.12
Theta range for data collection (°)	2.0 to 23.5	1.23 to 25.35	3.2 to 25.3	2.3 to 25.4
Index ranges	-13<h<17<br>-22<k<23<br>-23<l<18	-10<h<10<br>-19<k<13<br>-19<l<19	-10<h<8<br>-19<k<12<br>-19<l<18	-27<h<27<br>-9<k<11<br>-19<l<19
Reflections collected	35200	13435	13923	10720
Independent reflections	9616	3856	3901	3101
Completeness to theta	99.9	99.2	99.8	98.5
Max. and min. transmission	0.926 and 0.800	0.938 and 0.762	.859 and 0.663	1.000 and 0.776
Refinement method	full-matrix least-squares refinement on F^2	full-matrix least-squares refinement on F^2	full-matrix least-squares refinement on F^2	full-matrix least-squares refinement on F^2
Data/ restraints/ parameters	9616 / 1 / 577	3856 / 0 / 235	3901 / 0 / 235	3101 / 0 / 181
Goodness-of-fit on F2	0.726	1.077	0.874	0.974
R1 [I>2σ(I)]	0.0421	0.1122	0.0504	0.0428
R indices (all data)	R = 0.0713 and wR2 = 0.1400	R = 0.1809 and wR2 = 0.3803	R = 0.0695 and wR2 = 0.1546	R = 0.0621 and wR2 = 0.1007
Largest diff. peak and hole ($e.Å^{-3}$)	0.41 and -0.38	0.60 and -1.19	0.57 and -0.57	0.31 and – 0.48
Flack parameter	none	none	none	none
